# Littoral lichens as a novel source of potentially bioactive Actinobacteria

**DOI:** 10.1038/srep15839

**Published:** 2015-10-30

**Authors:** Delphine Parrot, Sanjay Antony-Babu, Laurent Intertaglia, Martin Grube, Sophie Tomasi, Marcelino T. Suzuki

**Affiliations:** 1 UMR CNRS 6226, Institut des Sciences chimiques de Rennes, Equipe PNSCM “Produits Naturels – Synthèses – Chimie Médicinale”, UFR Sciences Pharmaceutiques et Biologiques, Univ. Rennes 1, Université Européenne de Bretagne, 2 Avenue du Pr. Léon Bernard, F-35043 Rennes, France; 2 Sorbonne Universités; UPMC Univ. Paris VI, UMS 2348, USR 3579 LBBM, Observatoire Océanologique, Banyuls-sur-Mer 66650, France; 3 CNRS, USR 3579, LBBM, Observatoire Océanologique, F-66650, Banyuls/Mer, France; 4 CNRS, UMS 2348 (Plate-forme Bio2Mar), Observatoire Océanologique, F-66650 Banyuls/Mer, France; 5 Institut für Pflanzenwissenschaften Karl-Franzens-Universität Graz, Austria

## Abstract

Cultivable Actinobacteria are the largest source of microbially derived bioactive molecules. The high demand for novel antibiotics highlights the need for exploring novel sources of these bacteria. Microbial symbioses with sessile macro-organisms, known to contain bioactive compounds likely of bacterial origin, represent an interesting and underexplored source of Actinobacteria. We studied the diversity and potential for bioactive-metabolite production of Actinobacteria associated with two marine lichens (*Lichina confinis* and *L. pygmaea*; from intertidal and subtidal zones) and one littoral lichen (*Roccella fuciformis*; from supratidal zone) from the Brittany coast (France), as well as the terrestrial lichen *Collema auriforme* (from a riparian zone, Austria). A total of 247 bacterial strains were isolated using two selective media. Isolates were identified and clustered into 101 OTUs (98% identity) including 51 actinobacterial OTUs. The actinobacterial families observed were: Brevibacteriaceae, Cellulomonadaceae, Gordoniaceae, Micrococcaceae, Mycobacteriaceae, Nocardioidaceae, Promicromonosporaceae, Pseudonocardiaceae, Sanguibacteraceae and Streptomycetaceae. Interestingly, the diversity was most influenced by the selective media rather than lichen species or the level of lichen thallus association. The potential for bioactive-metabolite biosynthesis of the isolates was confirmed by screening genes coding for polyketide synthases types I and II. These results show that littoral lichens are a source of diverse potentially bioactive Actinobacteria.

Bioprospecting has traditionally been more successful for some prokaryotic groups than others^1^. Cultivable members of the Actinobacteria phylum in particular have an unrivalled track record as a major source of bioactive molecules (about 45% of all microbial bioactive products discovered^2^). Since the discovery of streptomycin, these strains have been isolated from various environments^3^. However, as the number of novel leads from Actinobacteria dwindled in the 1990 s^4^, the question was raised whether the “golden age” of actinobacterial bioprospecting was over. Increased research of environmental microorganisms from understudied ecosystems suggests this not be the case as new actinobacterial isolates continue to yield novel bioactive molecules.

So far most Actinobacteria have been isolated from terrestrial environments, especially soils^4^. However, marine environment might also be a promising source for bioprospecting^5^,^6^. Holding true to this promise, many novel chemical structures have been discovered from marine Actinobacteria^7^,^8^,^9^. Coastal systems, nonetheless, are still understudied and despite an early promising study from Watson and Williams (1974)^10^ more comprehensive actinobacterial surveys are pretty recent^11^,^12^. It should also be noted within this context, that these studies were mostly carried out on coastal sands and rhizosphere systems.

Among the underexplored coastal sources, marine lichens (referred to hereafter as those inhabiting the subtidal and intertidal zones) and littoral lichens (referred hereafter here as the those inhabiting the supratidal zone and subjected to sea spray) are unique. Like their terrestrial partners marine and littoral lichens are symbiotic associations between a photobiont (green algae and/or Cyanobacteria) and a mycobiont. Most lichens are outstanding producers of specific secondary metabolites that present biological activities e.g. antioxidant, cytotoxic, antimicrobial activities^13^,^14^,^15^,^16^,^17^. Recent studies have demonstrated the prevalence of lichen-associated bacteria^18^,^19^,^20^,^21^,^22^,^23^,^24^,^25^,^26^,^27^ including Actinobacteria^20^. While a number of publications reported the presence of cultivable bacterial associated with inland lichens, only few reported on cultivable bacteria from marine or littoral lichens (*Caloplaca verruculifera, Lecanora helicopis, Hydropunctaria maura* and *Verrucaria ceuthocarpa*)^28^,^29^ whereas the study on another littoral lichen *Roccella fuciformis* reported a lack of cultivable bacteria^20^. Thus, the diversity and the biological potential of lichen-associated bacterial communities are not yet sufficiently explored. In the study presented here, a culture-dependent approach was used to highlight marine and littoral lichens as a new source of cultivable bacteria and potential sources of Actinobacteria of interest.

## Results

### Overall diversity of isolates

**Figure 1:**
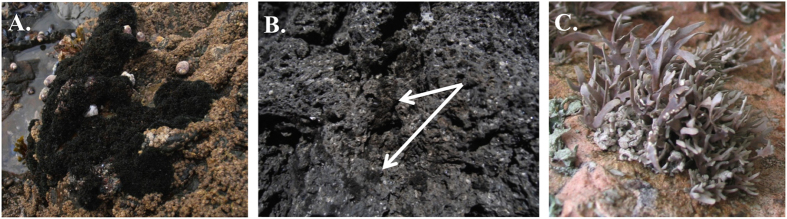
The lichens species studied. (**A**) *Lichina pygmaea* (black fruticose gelatinous cyanolichen on rocky shores), (**B**) *Lichina confinis* (black fruticose gelatinous cyanolichen on rocky shores, white arrows) and (**C**) *Roccella fuciformis* (grey fruticose lichen on sheltered vertical rockfaces).

After the lichen sampling (Fig. 1), serial dilutions of the washout and lichen homogenate (corresponding with bacteria on the surface and inside the thallus) were prepared. The bacterial inocula were plated on various media: marine agar (MA), actinomycete isolation agar (AIA) and International *Streptomyces* Project medium—2 (ISP 2) with nalidixic acid and cycloheximide (Table 1). Three representatives of each colony morphotype (whenever possible) on the triplicate plates were picked and transferred into the same media as they were isolated from. The partial 16S rRNA gene sequences of all strains were analyzed by comparison with the Eztaxon database using a global alignment algorithm^30^. The length of the sequenced fragments and the result of *BLASTn* comparisons are presented in Supplemental Table S1 with the similarity scores to the closest match.

Several non-actinobacterial strains were able to survive the nalidixic acid treatment and the isolation process failed to isolate bacteria for *L. pygmaea* from the wash suspension using AIA medium. All 16S rRNA gene sequences matched with entries in Eztaxon-server with similarities ranging from 91 to 100% (over around 800 bp). Overall, a total of 116 unique 16S rRNA sequence types were recovered with 38/55, 24/37 and 54/94 (unique 16S rRNA/total strains) retrieved from *Lichina confinis*, *L. pygmaea* and *R. fuciformis* respectively. Non-actinobacterial sequences were members of phyla Proteobacteria, Firmicutes and Bacteroidetes. Interestingly some of these sequences were quite novel as they had low sequence similarity (<95%) to known described species. Eighteen strains showed a similarity percentage below or equal to 96% suggesting putative new genera, e.g. MOLA1416 which presents a 91% 16S rRNA gene similarity with *Hoeflea phototrophica* (Supplemental Table S1).

We compared the 16S rRNA gene sequences from our study to those of another study targeting marine and littoral lichens^29^ and although this comparison is somewhat skewed due to our selective isolation method, we were able to identify a few highly similar (>99% over 90% of the sequence length) matches. Notably, these strains were similar to 1) the Alphaproteobacteria *Jannaschia pohangensis* (present in *L. confinis* and the Arctic lichens *C. verruculifera* and *H. maura*) and *Rhizorhapis suberifaciens* (present in *L. confinis* and the *C. verruculifera*) 2) the Actinobacteria *Streptomyces cyaneofuscatus* (present in all Brittany lichens and *L. helicopsis*), *Salinibacterium amurskyense* (present in *L. pygmaea* and *L. helicopsis*) and *Micrococcus luteus* (isolated from all lichens except for *L. helicopsis* and *H. maura*) and 3) the Firmicute *Bacillus aerius* (isolated from *L. pygmaea* and *H. maura*).

The results of the analysis by *BLASTn* using the parameters and thresholds described showed no overlap between isolates of the marine and costal lichens and the 2780 sequence in the queried database containing sequences from uncultured bacteria associated with lichens, while 8 strains from *Collema auriforme* (6 from wash water and 2 from homogenate) were similar (>98% identity over ca. 820 bp) to sequences belonging to Pseudomonadales (accession numbers JN023885, JN023697) previously retrieved from soils under moss crusts^31^.

### Diversity of Actinobacteria

**Figure 2:**
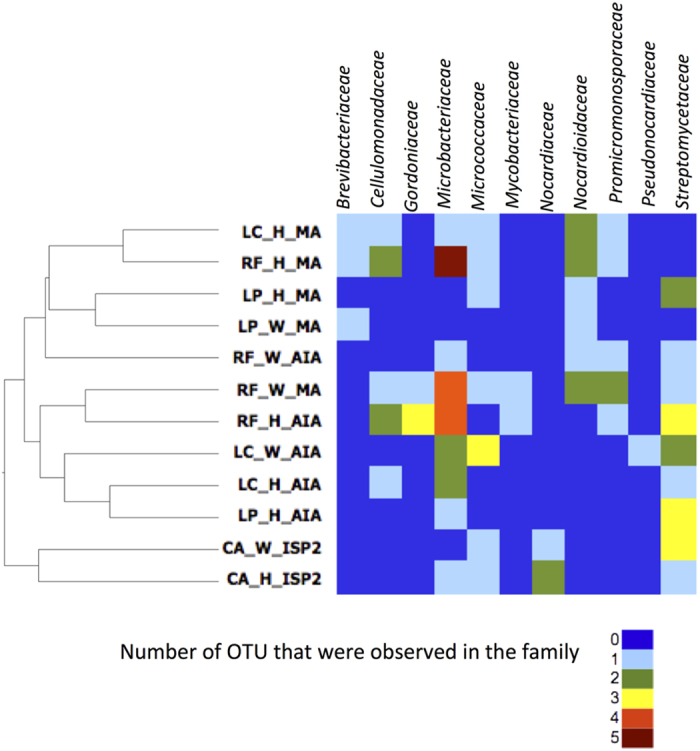
Clustering analysis (unweighted Unifrac distances) of 16S rRNA sequence from isolates affiliated to phylum Actinobacteria observed for the marine/littoral lichens *Lichina confinis*, *L. pygmaea* and *Roccella fuciformis* and the terrestrial lichen *Collema auriforme*. The accompanying heatmap shows the number of OTU observed from each actinobacterial family. Sample codes are described in Table 1.

In order to study the methodological and environmental factors that could influence the recoverability and diversity of Actinobacteria, a community analysis of sequences belonging to this phylum was performed by clustering sequences into OTUs at 98.5% identity (Supplemental Table S2). Since only a small fixed subset of representative strains from different morphotypes were recovered during isolation and subsequent subculture, presence and absence of OTUs were considered instead number of strains per OTU. In order to achieve a meaningful clustering using unifrac that utilizes phylogenetic distances, data from terrestrial lichen analyzed with a different medium was included as an outgroup. The clustering pattern is presented in Fig. 2.

Using the unweighted unifrac analysis (Fig. 2), bacterial communities could not be differentiated based on lichens species or sample treatment (e.g. homogenate or wash extract) but more reasonably by the media used. The communities associated with *Lichina* species showed a clustering depending on the isolation media whereas bacterial communities associated with *Roccella* species did not show such distinct clustering related to media. Overall, the three most evident clusters consisted of one corresponding to the communities isolated from MA medium, one from AIA medium and one specific from bacterial communities isolated from *C. auriforme* in ISP2 medium (Fig. 2).

We analyzed the two main clusters from marine lichens at a higher phylogenetic resolution. The heat-map at family level shows that Brevibacteriaceae, Nocardioidaceae and Promicromonosporaceae were mostly/primarily isolated in marine agar (MA medium) while Microbacteriaceae, Gordoniaceae, Pseudonocardiaceae and Streptomycetaceae were mostly frequent in actinomycetes isolation agar (AIA medium). Cellulomonadaceae and Mycobacteriaceae were isolated from both media. In just one case the most similar communities within a particular medium was from the same lichen genus (i.e. Microbacteriaceae and Streptomycetaceae in AIA medium for *Lichina* spp.; Fig. 2). It is important to point out that the heat-map is a reflection of the diversity (i.e. number of distinct taxa) within a family rather than a reflection of the number of strains. In addition we analyzed the environmental origin of nearest relatives of all Actinobacteria strains from marine/littoral lichens. Among the 55 top 16S rRNA sequences, 16 were from strains previously isolated from the marine environment (Table 2). In addition, except for strains related to *Paraoerskovia* and *Marmoricola* a clear relationship was not found between the high salinity medium utilized and the marine origin of nearest relatives, as many supposedly marine species were isolated in AIA and some supposedly non-marine organisms were isolated exclusively in MA. Nonetheless, it should be pointed out that among the 16 supposedly marine species retrieved 10 were exclusively isolated in MA.

The comparison of lichen bacterial communities using the same medium isolated from homogenate and washwater allowed a better view of the relationships between these communities and to infer a putative life style (epi- and/or endolichenic). Some bacterial families were mostly isolated from homogenate extract (Cellulomonadaceae and Streptomycetaceae) while Pseudonocardiaceae was only isolated from wash extract. Brevibacteriaceae, Micrococcaceae, Mycobacteriaceae, Nocardiaceae and Promicromonosporaceae were isolated from both sample types. Thereby bacterial communities associated with lichens might show a specific distribution in the lichen thallus (on the surface and/or inside; Fig. 2).

**Figure 3:**
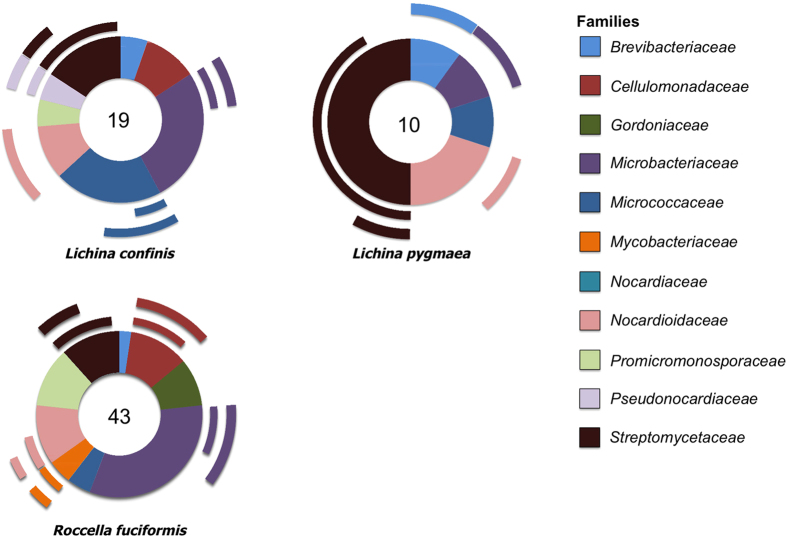
Doughnut charts showing proportions of actinobacterial OTUs observed in each family from the three different marine/maritime lichens studied. The two levels of ripples around the doughnuts represent the percentage of OTUs that were positive for genes coding the polyketide synthases. The inner ripple represents type I whereas the outer shows type II. Total number of strains are shown in the center of the doughnuts.

Figure 3 shows the overall Actinobacteria diversity observed in each lichen species irrespective of the sample kind (wash/homogenate) or the isolation media. It can be observed that *R. fuciformis* contained the most diverse communities, followed by *L. confinis* and *L. pygmaea*. This observation was however not true when four families of particular interest to bioprospecting, Nocardioidaceae, Promicromonosporaceae, Pseudonocardiaceae and Streptomycetaceae were considered. When comparing the Actinobacteria OTU richness among the two media a majority of bacterial communities was isolated from marine agar medium (54 versus 28) again indicating that salinity might be at play for the isolation of these bacteria. When a similar comparison of total OTU richness between homogenate and wash was done, a slight dominance of lichen-associated bacterial communities isolated from homogenate extract (48 versus 40) was recorded.

**Figure 4:**
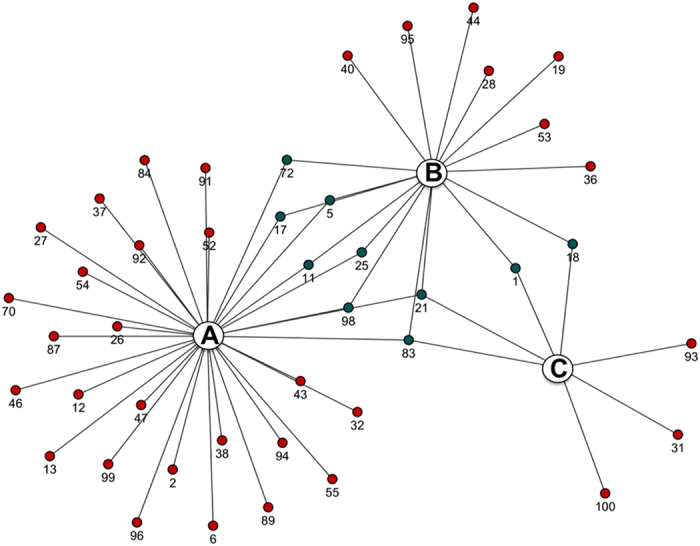
Cytoscape visualization of actinobacteria OTUs from (A) *R. fuciformis*, (B) *L. confinis* and (C) *L. pygmaea*. Green dots correspond to the common actinobacteria OTUs between lichen species.

At a higher phylogenetic resolution, each marine lichen species harbored specific actinobacterial communities. Two actinobacterial OTUs (operational taxonomic units) were common (OTU ID 1 and 18) (Supplemental Table S2) to *L. pygmaea* and *L. confinis* (Fig. 4) (similarity≥98.5%). Between marine and littoral lichens, six actinobacterial OTUs were common between *L. confinis* and *R. fuciformis* (OTU ID 5, 11, 17, 25, 72 and 98) while no actinobacterial OTUs was present only in *L. pygmaea* and *R. fuciformis*. Interestingly more similar actinobacterial strains were observed from lichens found spatially closer in the environment: *L. pygmaea* with *L. confinis* and *L. confinis* with *R. fuciformis*. Finally, two actinobacterial OTUs (OTU ID 21 and 83) corresponding to *Streptomyces* and *Micrococcus* genera were common to all three lichens (Fig. 4) and might represent eventual contaminants.

Regarding Actinobacteria from marine and littoral lichens, 8 strains (MOLA1448, MOLA1441, MOLA1513, MOLA1522, MOLA1523, MOLA1521, MOLA1599 and MOLA1557) representing 3 potential novel species were identified, based on a 16S rRNA gene similarity percentage less than 97% to type strains of described species (Supplemental Table S1, Table 2). These novel strains likely belong to the genera *Agromyces*, *Aeromicrobium*, and *Nocardioides*. Among these, 7 were isolated from marine agar media even though these are most closely related to bacterial strains isolated from terrestrial environments (Table 2). Five of the strains (MOLA1448 from *L. confinis*, MOLA1521-23 and MOLA1599 from *R. fuciformis*) belong to family Nocardioidaceae of interest for bioprospecting. Finally, as no previous chemical studies were conducted with the nearest relative of these five strains that potentially represent new species (Table 2), they would be of particular interest for the discovery of novel bioactive compounds.

### Genetic screening: PKS type I and II systems

The biotechnological potential of the isolates was examined using a PCR-based screening for PKS system types I and II. Type I was found to be more common found in 45.8% of the actinobacterial OTUs when compared to 34.7% that were positive for type II. However, the ratio was highly impacted by strains that belonged to the family Streptomycetaceae in which 92.8% were positive for PKS I and only 35% of those showed evidence of PKS II. One of the other three families of interest, Nocardioidaceae had more amplification of PKS II (50%) than type I (30%). Interestingly, Promicromonosporaceae strains were not positive for any of the genes tested (Fig. 3).

## Discussion

Several cultivable bacterial strains were isolated from marine and littoral lichens including members of the Firmicutes, Bacteroidetes, Proteobacteria and Actinobacteria. These cultivable strains belong to 30 different genera (Supplemental Table S1) some of which are known to produce bioactive compounds (Nocardiaceae, Promicromonosporaceae, Pseudonocardiaceae and Streptomycetaceae). The results of the *BLASTn* analysis against sequences of uncultured bacteria of lichen origin in the NCBI database suggests that, as is the case in the most other environments, the microorganisms isolated in our study did not represent a majority of the bacterial communities associated with lichens. However, to our knowledge to date only two studies have analyzed the microbial community of marine and littoral lichens (*Hydropunctaria maura, Ascophyllum nodosum, Caloplaca verruculifera, Verrucaria ceuthocarpa* and *Lecanora helicopis*) via cultivation independent methods^28^,^29^, the latter of which used community fingerprinting and did not report any 16S rRNA sequences from uncultured organisms. Hence, we cannot completely rule out that some of our isolates might represent dominant organisms in the marine and littoral lichens studied here, even though this is unlikely. On the other hand we were able to identify certain bacterial strains in at least three marine lichens from two distinct regions (*J. pohangensis*, *S. cyaneofuscatus* and *M. luteus*) indicating that these species might be frequently in association with marine lichens.

Interestingly many of the actinobacterial strains from marine and littoral lichens were not streptomycetes (Table 2). Not until recent times have the widespread occurrence and relative common presence of ”non-streptomycete” actinobacteria have only recently come to light^32^,^33^,^34^,^35^. The work presented here adds to the literature that once again indicates that the term “rare actinomycetes” is a misnomer^36^. These taxa (for example Nocardiaceae, Promicromonosporaceae, and Pseudonocardiaceae) have been already shown to be valuable bioresources for pharmacological prospecting. Furthermore, a large numbers of streptomycetes were also isolated from these lichens. Although members of this genus are often considered a “spent force” in terms of biodiscovery, one has to note that at least 13 novel biomolecules have been reported from this taxa in the first half of 2014 alone^37^,^38^,^39^,^40^,^41^,^42^,^43^,^44^,^45^,^46^,^47^,^48^. Additionally, Takagi and Shin-ya (2011)^49^ have shown novelty in actinobacterial taxa alone does not always mean the presence of novel bioactive compounds.

Overall, many of the strains were closely related and in some cases had 16S rRNA genes identical to strains know to produce bioactive compounds in the genera *Streptomyces*, *Mycobacterium*, *Curtobacter*, *Microbacterium*, *Micrococcus* and *Nocardioides*. More specifically, 41 different compounds have been reported for strains or species most closely related to our actinobacterial isolates (15 compounds described from bacterial strains isolated from marine environments and 26 compounds for bacteria isolated from other environments; Table 2) those include exopolysaccharides, diketopiperazines, angucyclines, anthracyclines, a macrolactone, and an enediyne). The biosynthesis of many of these compounds include polyketide synthases and in prokaryotes, polyketide synthases of types I, II and III are present. However, type I PKS systems are much more common, specific for bacteria, and mainly found in actinomycetes where some are known to produce bioactive compounds^50^. The presence of PKS types I and II gene clusters in marine and littoral lichen isolates, furthers the evidence of their biosynthetic potential.

The use of two different media for marine and littoral lichens clearly increased the diversity of Actinobacteria that could be isolated. In addition even with the addition of nalidixic acid, marine agar (MA) medium allowed the isolation of a wider diversity of bacteria in the Proteobacteria Firmicutes and Bacteroidetes and almost the 2/3 of the unique bacterial strains from *L. confinis* were isolated using MA medium (Supplemental Table S1). The choice of growth media influenced the bacterial diversity associated with lichens more than the sample type indicating that it is an important factor to consider to increase the total cultivable diversity associated with various organisms and that it is the use of a wider range of media in isolation efforts, is likely to increase overall isolate diversity. Our results also indicate that some of the bacterial families could be more strongly associated with the lichen thallus. For instance bacteria belonging to the family Cellulomonadaceae, Microbacteriaceae, Gordoniaceae and Streptomycetaceae were more predominant in homogenate samples, thus indicating a putatively closer association of these taxa. Whereas presence of Pseudonocardiaceae strains in only one of the wash samples might indicate an episymbiotic association or a more occasional relationship.

Some of the bacterial strains associated with lichens, have been previously investigated for the production of secondary metabolites and some interesting compounds have been described. Uncialamycin an enediyne^51^ with antibacterial properties against human pathogens (*Staphylococcus aureus*, *Escherichia coli* and *Burkholderia cepacia*), and cytotoxic properties, as well as cytotoxic cladoniamides A-G, were isolated from *Streptomyces uncialis* associated with *Cladonia uncialis*^51^,^52^. Angucycline with cytotoxic and antibacterial properties against *Micrococcus luteus* and a butenolide (inactive) were isolated from a *Streptomyces* sp. associated with an unidentified lichen collected in Japan^53^. Six aminocoumarins (coumabiocines A-E) showing antibacterial properties were also isolated from a *Streptomyces* sp. associated with *Cladonia gracilis*^54^. Thus bacteria associated with lichens show interesting biological properties in terms of producing antibacterial or DNA damaging molecules. The study presented here adds to these observations and shows that the lichens and in particular marine/littoral lichens are not only a source of *Streptomyces* strains but can also be a source of more Actinobacteria with high potential for the exploitation of bioactive compounds. Marine and littoral lichen-associated bacteria represent thus a promising yet under explored to discover new natural products.

A major aspect of bacterial symbionts is their possible interactions with their hosts. Lichens are unique models to study interactions between fungi, algae and bacterial symbionts^22^. One important approach to understand such complex and yet interesting interactions is to focus on interactions from isolates retrieved from the lichen holobiont. This is particularly true for chemical interactions since currently access of specific member of the symbiosis is not possible without cultivation. Actinobacteria are especially known to influence secondary metabolism of fungi^55^ and thus these bacteria are good model organisms to study metabolic interactions. Although, as we showed here, culture-dependent methods do not reflect the prevalent organisms, in the context of chemical interactions these “rare” microorganisms could in fact be more relevant than their abundance alone might indicate. Culture based approaches such as the one used in this work are therefore necessary to advance the overall understanding of interactions among different organisms in these symbiosis.

## Methods

### Lichen sampling

Lichen samples were collected from France at Erquy (48°37′47.9′′ N and 2°28′31.55′′ W) in April 2012 and from Austria at Kesselfallklamm (47°12′21.26′′ N and 15°23′57.27′′ E) near Graz in November 2012. Three marine/littoral species were collected on seashore rocks on Brittany coasts (France): *Lichina pygmaea* (Müll.) Agardh (marine lichen), *L. confinis* (Lightf.)Agardh., (marine lichen) and *Roccella fuciformis* (L.) DC., (littoral lichen) and, one inland species was collected on rocks (Austria): *Collema auriforme* (With.). Lichens were identified based on their morphological characters such as size, color and chemical reaction (potassium hydroxide, *para*-phenylenediamine, sodium hypochlorite).

### Isolation of cultivable bacteria

Marine and littoral lichens were briefly washed with sterile water in sampling sites (to remove non-symbiotic bacteria) then kept on ice through transit to the lab. On arrival to the lab the samples were aseptically divided into small pieces (1–2 g) using sterile scalpels. The pieces were washed three times with 20 ml of sterile seawater. The wash suspensions were stored and the washed lichen pieces were ground using a blender. The wash suspensions and lichen homogenates were used as separate inocula. Serial dilutions (N to N^−3^) in sterile seawater of wash and homogenate were performed and 100 μL were plated in Marine Agar (MA, Difco^TM^ Marine Agar, BD Le Pont de Claix, France) and Actinomycete Isolation Agar (AIA, Difco^TM^ Actinomycete Isolation Agar, BD) both media supplemented with nalidixic acid [(NA382-1G) Sigma-Aldrich, Lyon, France] and cycloheximide [(C7698-5G) Sigma-Aldrich] in triplicate. Plates were incubated at 25 °C until the growth of the colonies on the Petri dishes and until the no new colonies appeared (up to 21 days). Colonies were isolated and purified using morphological characteristics [color, diameter, surface (smooth and/or rough), relief (convex or flat) and edge format (regular or irregular)], on their respective media (MA or AIA). Bacterial strains were stored in a 50% glycerol solution and 5% DMSO in Marine Broth (MB, Difco^TM^, BD) at −80 °C. The strains are deposited in MOLA culture collection of the Observatoire Oceanologique de Banyuls/Mer. Bacteria from *C. auriforme*, which was used as an outgroup for the analysis of marine/littoral lichens were isolated using a similar protocol, except that lichens were washed with NaCl (0.85%)/Peptone from casein (1%) solution and plated into Difco^TM^ ISP 2 agar (BD).

### Genomic DNA extraction

Colonies were picked using sterilized inoculating loop and transferred in their respective liquid media depending on the original isolation media. That is, strains isolated from marine agar were cultured in marine broth (MB) and those from actinomycetes isolation agar were enriched in Luria-Bertani broth (LB) for 72 hours at 25 °C. Suspensions of 950 μL of these cultures were dispensed in microcentrifuge tubes. The tubes were centrifuged at 12500 g for 5 min and supernatants were discarded. The pelleted biomass was used to isolate genomic DNA using Wizard^®^ Genomic DNA Purification Kit (Promega, Lyon, France) following the manufacturer’s protocol.

### PCR amplification of 16S rRNA genes

Aliquots (1 to 2 μL) of DNA samples were used as templates to amplify the 16S rRNA gene in a 10 μL PCR mixture with 0.4 μL (10 μM) universal bacterial primers 27Fmod (5′-AGR GTT TGA T CM TGG CTC AG-3′^56^ and 1492Rmod (5′-TAC GGY TAC CTT GTT AYG ACT T-3′^57^, 1X buffer, 1 μL MgCl_2_ (25 mM), 0.4 μL dNTPs (20 mM) and 0.05 μL of Platinum *Taq* Polymerase (5 U/μL; Life Technologies, St Aubin, France).PCRs were conducted in a Veriti Thermal cycler (Life Technologies) with initial denaturing step (95 °C for 5 min) followed by 35 cycles of denaturation at 95 °C for 30 sec, primer annealing at 50 °C for 30 sec and primer extension step at 72 °C for 1.30 min, and a final extension step was at 72 °C for 10 min. Amplicons were separated by agarose gel electrophoresis (1%, 15 min at 100 V) in TAE buffer stained with ethidium bromide and visualized under UV.

### Sequencing and phylogenetic analysis of 16S rRNA gene products

The amplicons were purified using the Agencourt^®^AMPure^®^ XP Kit (Beckman Coulter, Villepinte, France). Aliquots (1 μL) of these samples were used as templates in a 10 μL sequencing reaction mixture with 0.5 μL Big Dye Terminator (V3.1; Life Technologies), 1.75 μL of Buffer BDT (5X; Life Technologies) and 1 μL (3.2 μM) universal bacterial primer 907r (5′-AGR GTT TGA TCM TGG CTC AG-3′). The dye terminator reactions were conducted in a Verity Thermal cycler (Life Technologies) with 40 cycles of 95 °C for 10 sec, 50 °C for 5 sec and 55 °C for 2.30 sec. The post-reaction mixes were cleaned using Agencourt^®^ CleanSeq^®^ Kit (Beckman Coulter) and sequenced using ABI 3130xl genetic Analyser Sequencer (Life Technologies). The partial 16S rRNA gene sequences obtained were aligned using the Staden Package (Gap4). However, 19 samples of bacteria from *C. auriforme* were purified using NucleoSpin^®^ Gel and PCR Clean-up Kit (Macherey-Nagel Dueren, Germany) and sequenced by Macrogen (Amsterdam, The Netherlands) using universal bacterial primer 907r also. All sequences were compared using the BLAST algorithm with the sequences of EzTaxon server (http://eztaxon-e.ezbiocloud.net) database^30^. Sequences were deposited in Genbank under accession numbers KM273865—KM274111.

### Community analysis of cultured Actinobacteria

The sequences of all isolates were subject to an analysis pipeline using QIIME v1.5.0^58^. All sequences were clustered into at 98.5% identity level using the uclust algorithm in usearch 5.2 (http://drive5.com/usearch/). OTUs were classified using the rdp_classifier and a modified database based on the Green genes October 2012 taxonomy (http://greengenes.secondgenome.com). An OTU table considering sampling units of unique combinations between lichen species, inoculum (homogenate or wash water) and isolation medium was created. Since by the addition of nalidixic acid the study selected for Actinobacteria, the subsequent analysis was performed only with organisms in this phylum. The OTU table and sequence files were parsed using shell scripts to select Actinobacteria. Sequences were aligned and masked to enable reconstruction of phylogenetic tree using QIIME’s default parameters (a pynast alignment and a fast tree tree). The placement of all sequences in the tree was used to calculate an unweighted unifrac dissimilarity matrix which was finally used for UPGMA clustering and Cytoscape (v.3.1.0) visualization.

### BLASTn analysis

In order to compare isolate sequences to those most frequently found in lichens via cultivation independent methods we performed a NCBI Genbank search using with “[lichen OR lichens] AND 16S AND uncultured NOT photobiont” as query. All the resultant 2780 sequences were downloaded and used to create a database for blast queries. Sequences of all isolates were used as queries to the database using *BLASTn* v. 2.2.22^59^ with the following parameters: -e 1 -b 10 -v 10 -n T -r 1 -q -2 -G 0 -E 0 -m 8. Sequences with identities of above 98% over 200 bp were identified by parsing using awk and shell scripts. In addition in order to identify we performed a megablast analysis against the NCBI Genbank database (using default parameters) and retrieved the top scores for from *cultured* microorganisms. The environmental origin of the organisms was determined based on a search in the databases of the culture collections where these strains were deposited or the metadata associated with the NCBI entry. Data regarding the production of biomolecules was obtained by searches in the Web of Science, (http://apps.webofknowledge.com) or the REAXYS (http://www.reaxys.com) Natural Products databases. Finally in order to compare our strains to the strains isolated from those isolated by Sirgubjörnsdòttir and colleagues from other marine lichens^29^, sequences from that study were retrieved and used to create a local blast database which was queried using megablast.

### Screening for presence of PKS type I and II systems

In the lookout for evidence of biosynthetic potential, PCR based screening was carried out on genomic DNA isolated from the strains. PKS-I amplifications were carried out using the primers set-2 F (5′-CCS CAG SAG CGC STS TTS CTS GA-3′) and set-2 R (5′-GTS CCS GTS CCG TGS GTS TCS A-3′) as described by Courtois *et al*., (2003)^60^. The original conditions described by the authors were modified so as to accommodate use of fast PCR reaction mix KAPA2G (Clinisciences, Nanterre France). Briefly, the amplifications were performed using 4 min initial denaturation at 95 °C followed by 4 touch-down cycles with annealing temperature of 65–62 °C for 15 sec. The touch-down cycles were followed by 36 amplification cycles of constant annealing temperature of 61 °C for 15 sec. Both the touch-down and the following standard cycles used a denaturation temperature of 95 °C for 15 sec and amplification at 72 °C for 30 sec. Cycling was followed by a final amplification step at 72 °C for one minute.

PKS type II screens were carried out using the primers KSαF (5′-TSG CST GCT TCG AYG CSA TC-3′) and KSαR (5′-TCG CCB AAG CCN AAG GT-3′) as described by Metsä-Ketelä *et al*., (1999)^61^. The modified PCR conditions were as follows: the initial denaturation was carried out at 95 °C for 4 minutes followed by 40 cycles of denaturation at 95 °C for 15 sec, annealing from 56 to 64 °C for 15 sec and amplification at 72 °C for 30 sec. The PCR reaction concluded with a 1 min extension at 72 °C. The potential presence of the polyketide synthase systems were inferred based on production of amplicons of desired sizes (ca. 600 bp).

## Additional Information

**How to cite this article**: Parrot, D. *et al*. Littoral lichens as a novel source of potentially bioactive Actinobacteria. *Sci. Rep*. **5**, 15839; doi: 10.1038/srep15839 (2015).

## Supplementary Material

Supplementary Information

## Figures and Tables

**Table 1:** Description of the lichen samples used as inocula.

Lichen	Symbiont	Environment	Extract	Medium^1^	Description^2^
*L. confinis*	Cyanobacteria	Marine	Homogenate	MA	LC_H_MA
*L. confinis*	Cyanobacteria	Marine	Wash	AIA	LC_W_AIA
*L .confinis*	Cyanobacteria	Marine	Homogenate	AIA	LC_H_AIA
*L. confinis*	Cyanobacteria	Marine	Wash	MA	LC_W_MA
*L. pygmaea*	Cyanobacteria	Marine	Homogenate	MA	LP_H_MA
*L. pygmaea*	Cyanobacteria	Marine	Homogenate	AIA	LP_H_AIA
*L. pygmaea*	Cyanobacteria	Marine	Wash	MA	LP_W_MA
*R. fuciformis*	Green algae	Maritime	Homogenate	MA	RF_H_MA
*R. fuciformis*	Green algae	Maritime	Wash	AIA	RF_W_AIA
*R. fuciformis*	Green algae	Maritime	Homogenate	AIA	RF_H_AIA
*R. fuciformis*	Green algae	Maritime	Wash	MA	RF_W_MA
*C. auriforme*	Cyanobacteria	Inland	Wash	ISP2	CA_W_ISP2
*C. auriforme*	Cyanobacteria	Inland	Homogenate	ISP2	CA_H_ISP2

^1^Culture media used with MA: Marine agar media, AIA: Actinomycetes Isolation Agar media and ISP2: International Streptomyces Project 2 agar media.

^2^Description correspond to the lichen sample (LC: *L. confinis*, LP: *L. pygmaea*, RF: *R. fuciformis* or CA: *C. auriforme*), the extract origin (H: Homogenate and W: Wash) and the culture media (MA, AIA or ISP2).

**Table 2:** List of cultivable Actinobacteria isolated from marine and littoral lichens.

Strains	Media	Top HitsNCBI	Scores	Identity (%)	Strains and Origin	Description	Production of metabolites
MOLA1487MOLA1509	AIAAIA	NR116119.1	1548	100	Strain YIM60513^T^ Endophyte of *Gloriosa superba*	*Saccharopolyspora gloriosae*	NF
MOLA1503MOLA1508	AIAAIA	HQ113380.1 NR037048.1 AB013920.1	1493	99	Strains JCM10270^T^*; Kitami A1*, DD4 digestive tract of *Daphnia magna ;* wastewater of a sugar-beet factory	*Microbacterium kitamiense (Microbacterium aurantiacum)*	*Exopolysaccharides^62^
MOLA1515MOLA1570	AIAMA	EF672652.1	1391 1408	97	Strain AR33 rhizosphere of heavy metal accumulating willow trees	*Agromyce s*sp. *(Agromyces terreus)*	NF
MOLA1512MOLA1526MOLA1527MOLA1528MOLA1533MOLA1534MOLA1536MOLA1554MOLA1555MOLA1574MOLA1585MOLA1592MOLA1595MOLA1602MOLA1616	AIAMAMAMAMAMAMAMAMAMAAIAAIAMAAIAAIA	AB695377. NR114322.1	1448–1511	99	Strains H97-3^T^, H83-5, isolated from sea sediment	*Sediminihabitans luteus*	NF
MOLA1486	AIA	KJ627769.1 KC768764.1 FR744933.1 AB330407.1	1531	100	Strains JI2, Iso-74, BAM270, CC0524 oil filed water, water treatment sand filter, soil, Baltic sea	*Microbacterium oxydans Microbacterium* sp. *(Microbacterium foliorum*†)	†Volatile sulfur and non-sulfur compounds (Str. C45)^63^
MOLA1420MOLA1421MOLA1427MOLA1488MOLA1493MOLA1578MOLA1596MOLA1615MOLA1611MOLA1612MOLA1614	MAMAAIAAIAAIAAIAAIAAIAAIAAIAAIA	HG965212.1 KJ676478.1 JX013966.1	1489–1543	99–100	Strains M27*, NEAE-42, sj32 Intertidal seaweed *Fucus spiralis*, soil	*Streptomyces cyaneofuscatus Streptomyces cavourensis Streptomyces flavolimosum (Streptomyces cyaneofuscatus)*	*daunomycin, *cosmomycin B *galtamycin B *maltophilin^64^
MOLA1492	AIA	JX173791.1	1522	100	Strain TRM46621 “salty beach”, Xinjiang	*Streptomyces* sp. (*Streptomyces carpaticus)*	NF
MOLA1491	AIA	KM978822.1	1515	99	Strain AHT-1 River sediment	*Kocuria rhizophila*	NF
MOLA1490MOLA1450	AIAMA	FJ015034.1	1480	99	Strain M52-4.1 turbot larval rearing unit, tank surface	*Micrococcus* sp. (Micrococcus luteus*)	*Lutoside (unnamed strain)^65^ *[3H]-Indole acetic acid (unnamed strain)^66^
MOLA1489	AIA	KF591409.1	1230	99	Strain PK3 bark of ginkgo	*Arthrobacter sp*.(*Tersicoccus phoenicis*)	NF
MOLA1484	AIA	JQ924405.1	1500	100	Strain CGMCC 4.1782^T^ Potato spot	*Streptomyces praecox* (*Streptomyces setonii*†)	Diketopiperazines (Str. 291–11)^67^ †FR109615^68^ †16-Deethylindanomycin^69^ †5-Hydroxymethylblasticidin S and blasticidin S (Culture A83094)^70^
MOLA1447	MA	NR_114323.1	1472	99	Strain H25-14^T^ Sea sediment	*Paraoerskovia sediminicola*	NF
MOLA1519MOLA1520MOLA1544MOLA1553MOLA1561MOLA1572MOLA1573	MAMAMAMAMAMAMA	JQ716239.1	1430–1452	98	Strain BJGMM-B34 Soil samples from the Yellow River Delta	*Cellulosimicrobium* sp. (*Cellulosimicrobium terreum*)	NF
MOLA1521MOLA1522MOLA1523MOLA1599	MAMAMAMA	AB461094.1	1380–1404	97	Strain IK2_56P soybean stem	*Nocardioidaceae bacterium (Nocardioides mesophilus)*	NF
MOLA 1448 MOLA1433MOLA1434MOLA1446	MAMAMAMA	DQ448721.1	1454–1461	98	Strain CNJ872 PL04 marine sediment	*Marmoricola* sp*. (Marmoricola aequoreus)*	NF
MOLA1418MOLA1445	MAMA	KJ843153.1	1504–1548	100	Strain VL-80 onion waste	Micrococcus luteus* *(Micrococcus aloeverae)*	*Lutoside (unnamed strain)^65^ * [3H]-Indole acetic acid (unnamed strain)^66^
MOLA1444MOLA1565	MAMA	KM817772.1	1450–1489	99	Strain SKCB-14 sorghum	*Brevibacterium* sp. *(Brevibacterium epidermidis)*	NF
MOLA1441MOLA1513	MAAIA	NR_116877.1	1341	95	Strain BZ41^T^ isolated from hydrocarbon-contaminated soil	*Agromyces bauzanensis (Agromyces terreus)*	NF
MOLA1435	MA	HQ677231.1	1504	99	Strain A-08 in larval culture of *Argopecten purpuratus*	*Brevibacterium* sp. *(Brevibacterium picturae)*	NF
MOLA1425MOLA1405	MAAIA	LN626361.1	1531	100	Strain M-26 *Fucusspiralis*(host)	*Streptomyces albidoflavus*†	†Albaflavenone (Str. DSM 5415)^71^†Antimycin A18 (Str. I07A–01824)^72^ †Dibutylphthalate (str MTCC 3662)^73^
MOLA1408	AIA	KF848947.1	1522	100	Strain FZ3 hazelnut husk waste	*Streptomyces* sp. *(Streptomyces rubiginosohelvolus*†)	†Rubomycin^74^
MOLA1407	AIA	HM584291.1	1535	100	Strain CJ-G-TSA6 isolated from internal organs of edible snow crabs (*Chionocetes japonicus*)	*Agreia* sp. *(Salinibacterium amurskyense)*	NF
MOLA1406	AIA	KM678243.1	1522	100	Strain B035 sediment from Lake Michigan	*Streptomyces flavogriseus* †*(Streptomyces anulatus* †§)	† Bromoxantholipin (Str. SIIA-A02191)^75^ Clavulanic acid (Str. ATCC 33331)^76^ §valinomycin, montanastatin^77^ (Str. Montana, Str. Malaysia) † telomestatin (Str. 3533-SV4)^78^ †phenazine-1-carboxylic acid , endophenazines A-D (Str. 9663)^79^ (trimethylglucosaminium)chitotriomycin (Str NBRC 13369)^80^ † Glutarimide derivative^81^ †Dihydroabikoviromycin^82^
MOLA1548	MA	AJ296094.1	1395	97	Strain OS-6 isolated from coastal marsh	*Microbacterium* sp. *(Microbacterium murale)*	NF
MOLA1545	MA	AB376081.1	1531	100	Strain YT0066 “environmental samples”	*Microbacterium* sp. *(Microbacterium pumilum)*	NF
MOLA1538	MA	JF274912.1	1404	97	Strain PL34a1_S1 olive-mill wastewater	*Aeromicrobium* sp. *(Aeromicrobium tamlense)*	NF
MOLA1530	MA	AF544638.1	1509	99	Strain VM0587 PAH-contaminated soil	*Mycobacterium vaccae* § *(Mycobacterium vanbaalenii)*	§ Poly-α-(1- > 4)-3-*O*-methyl-D-mannopyranose (Str. ATCC 15483)^83^
MOLA1524MOLA1525	MAMA	FR682685.1	1504	99	Strain R-36360 soil	*Microbacterium* sp. *(Microbacterium invictum)*	NF
MOLA1516	MA	NR_112842.1	1502	99	Strain Sp080513SC-30^T^* marine sponge *Haliclona* sp.	*Streptomyces tateyamensis*	*JBIR-107^84^
MOLA1597	AIA	NR_043931.1	1489	99	Strain UMS-62 rhizospheric soil of *Aliumvictorialis* var. *platyphyllum*	*Agromyces allii (Agromyces terreus)*	NF
MOLA1593	AIA	JX949623.1	1312	97	Strain MDT1-31-2 glacier	*Gordonia* sp. *(Gordonia hankookensis)*	NF
MOLA1590	AIA	KF561998.1	1539	100	Strain M7ER1 Sunflower	*Microbacterium* sp. *(Microbacterium paraoxydans)*	NF
MOLA1589	AIA	HE716916.1	1537	100	StrainJSM-04 *Miscanthus* sp.	*Curtobacterium flaccumfaciens*	§ Carotenoid glycosides Cp. 450, C-460, Cp473 (Str. DSM 20149)^85^
MOLA1588	AIA	KJ855063.1	1275	98	Strain QIA-38 (*Bos taurus coreanae*	*Mycobacterium* sp. *(Mycobacterium flavescens)*	NF
MOLA1584MOLA1594	AIAAIA	EU584521.1	1487	100	Strain Everest-gws-50 glacial meltwater (Mount Everest)	*Microbacterium* sp. (Microbacterium kitamiense*)	*Exopolysaccharides^62^
MOLA1583MOLA1591MOLA1587MOLA1586	AIAAIAAIAAIA	NR_104507.1	1517	99	Strain ON-33^T^ soil around a wastewater treatment	*Gordonia hankookensis*	NF
MOLA1582MOLA1531	AIAMA	EU741242.1	1454	99	Strain 13679F Marine beach sand	*Gordonia* sp. *(Gordonia defluvii)*	NF
MOLA1580	AIA	KC355272.1	1524	99	Strain KUDC1765 Rhizosphere *Elymus tsukushiensis*	*Isoptericola variabilis (Isoptericola nanjingensis)*	NF
MOLA1579	AIA	FR837628.1	1507	99	Strain 5-4-1 oligotrophic peat soil	*Streptomyces beijiangensis (Streptomyces brevispora)*	NF
MOLA1537MOLA1546MOLA1549MOLA1558MOLA1559MOLA1563MOLA1576	MAMAMAMAMAMAMA	NR_112793.1 NR_114324.1	1459 1478	98 99	CTT-37^T^ = NBRC 104352^T^ sea sediment	*Paraoerskovia marina (Sediminihabitans luteus Paraoerskovia sediminicola)*	NF
MOLA1575MOLA1608	MAMA	AY159888.1	1535	99	Strain 98TH11321 biofilm forming marine bacteria on glass surface in Dae-Ho Dike (Korea)	*Micrococcus* sp. *(Micrococcus yunnanensis*†)	† Carotenoids Sarcinaxanthin, Sarcinaxanthin Monoglucoside, Sarcinaxanthin Diglucoside^86^
MOLA1569	MA	HQ219671.1	1541	100	Strain AP01 soil	“Bacterium” *(Brevibacterium epidermidis)*	NF
MOLA1567MOLA1566MOLA1564	MAMAMA	AB646581.2	1443	98	Strain SL10 polluted tropical soil	*Microbacterium* sp. *(Microbacterium oleivorans)*	NF
MOLA1562	MA	AB563787.1	1454	98	Strain JCM 9634 Soil, Hyogo Pref., Japan	*Microbacterium* sp. *(Microbacterium suwonense)*	NF
MOLA1560	MA	NR 114323.1	1474	99	Strain H25-14^T^ sea sediment	*Paraoerskovia sediminicola*	NF
MOLA1557	MA	DQ180951.1	1461	99	Strain MI-59a Kartchner Caverns in Benson	*Nocardioides* sp. *(Mumia flava)*	NF
MOLA1556MOLA1550MOLA1541MOLA1571	MAMAMAMA	NR_042708.1	1526	99	Strain DC-200^T^ homemade compost	*Microbacterium invictum*	NF
MOLA1517MOLA1518MOLA1532MOLA1605	MAMAMAAIA	KC355274.1	1509	99	Strain KUDC1767 rhizosphere	*Isoptericola variabilis (Isoptericola nanjingensis)*	NF
MOLA1603	AIA	EU908199.1	1513	99	Strain MS218 deep sea mud in South China sea	*Streptomyces* sp. *(Streptomyces drozdowiczii*§)	§ Marformycins A-F (str. SCSIO 10141)^87^
MOLA1601MOLA1598	AIAMA	NR_044184.1	1537	99	Strain DS-10^T^ soil	*Agromyces terreus*	NF
MOLA1600MOLA1610	AIAAIA	EU876699.1	1528	99	Strain M2004 rhizosphere soil	*Streptomyces* sp. *(Streptomyces atroolivaceus*†§)	† Leinamycin (Strain sv)^88^ † Mikamycin A (strain S140)^88^ § Berninamycins A and E (strain NBRC 12741^T^)^89^
MOLA1606	MA	KC213957.1	1358	98	Strain S_S_TSA_8 roach gut	*Microbacterium pumilum (Microbacterium saccharophilum)*	NF
MOLA1617	AIA	KP170480.1	1221	99	Strain VLK-10 soil	*Streptomyces albiaxialis*	NF
MOLA1568	MA	DQ448693.1	1531	100	Strain CNJ737 marine sediment	*Brevibacterium* sp *(Brevibacterium epidermidis)*	NF
MOLA1607	AIA	JN896615.1	1544	99	Strain FMN08 ND	*Nocardioides* sp. *(Nocardioides albus*§†)	§ Teichoic acid (strain VKM Ac-805(T))^90^ † Leucylblaticidin, Rodaplutin (Strains DSM 3176, DSM 3177)^91^

Strains: strains from the MOLA collection of microorganisms (WDCM911); Media: MA (marine agar) and AIA (Actinomycete Isolation Agar); Top Hits NCBI: accession number of NCBI sequences with top *blastn* scores; Scores (NCBI *blastn* scores); description: NCBI description and in parenthesis top EZTaxon type species hit whenever different (see also table S1); Production of metabolites. Description based on literature searches (Web of Science, http://apps.webofknowledge.com) or queries against the Natural Products in the REAXYS (http://www.reaxys.com) databases. Strains shaded in grey had highest hits to strains previously recovered from marine environments. ND: not determinated and NF: not found. *correspondence between metabolite and strain †found in a strain without a 16S rRNA sequence; § found in a strain with a different 16S rRNA sequence than the top NCBI hit; ¶ found in a different strain with identical 16S rRNA sequence than the top NCBI hit.

## References

[b1] Demain A. L. Importance of microbial natural products and the need to revitalize their discovery. J. Ind. Microbiol. Biotechnol. 41, 185–201 (2014). 23990168 10.1007/s10295-013-1325-z

[b2] Jose P. A., Robinson S. & Jebakumar D. Non-streptomycete actinomycetes nourish the current microbial antibiotic drug discovery. Front. Microbiol. 4, 2008–2010 (2013). 10.3389/fmicb.2013.00240 PMC3747354 23970883

[b3] Koehn F. E. & Carter G. T. The evolving role of natural products in drug discovery. Nat. Rev. Drug Discov. 4, 206–20 (2005). 15729362 10.1038/nrd1657

[b4] Bérdy J. Bioactive microbial metabolites. J. Antibiot. (Tokyo). 58, 1–26 (2005). 15813176 10.1038/ja.2005.1

[b5] Bull A. T., Stach J. E. M., Ward A. C. & Goodfellow M. Marine actinobacteria: perspectives, challenges, future directions. Antonie Van Leeuwenhoek 87, 65–79 (2005). 15971359

[b6] Fiedler H.-P. . Marine actinomycetes as a source of novel secondary metabolites. Antonie Van Leeuwenhoek 87, 37–42 (2005). 15726289 10.1007/s10482-004-6538-8

[b7] Fu P., Johnson M., Chen H., Posner B.A. & Macmillan & J. B. Carpatamides A-C, Cytotoxic Arylamine Derivatives from a Marine-Derived Streptomyces sp. J. Nat. Prod. 77, 1245–1248 (2014). 24754815 10.1021/np500207p PMC4035114

[b8] Goodfellow M. . Verrucosispora maris sp. nov., a novel deep-sea actinomycete isolated from a marine sediment which produces abyssomicins. Antonie Van Leeuwenhoek 101, 185–93 (2012). 21989685 10.1007/s10482-011-9651-5

[b9] Oh D.-C., Williams P. G., Kauffman C. a, Jensen P. R. & Fenical W. Cyanosporasides A and B, chloro- and cyano-cyclopenta[a]indene glycosides from the marine actinomycete ‘Salinispora pacifica’. Org. Lett. 8, 1021–4 (2006). 16524258 10.1021/ol052686b

[b10] Watson E. T. & Williams S. T. Studies on the ecology of actinomycetes in soil—VII. Actinomycetes in a coastal sand belt. Soil biol. Biochem. 6, 43–52 (1974).

[b11] Antony-Babu S., Stach J. E. M. & Goodfellow M. Genetic and phenotypic evidence for Streptomyces griseus ecovars isolated from a beach and dune sand system. Antonie Van Leeuwenhoek 94, 63–74 (2008). 18491216 10.1007/s10482-008-9246-y

[b12] Hong K. . Actinomycetes for marine drug discovery isolated from mangrove soils and plants in China. Mar. Drugs 7, 24–44 (2009). 19370169 10.3390/md7010024 PMC2666887

[b13] Lauterwein M., Oethinger M., Belsner K., Peters T. & Marre R. *In Vitro* Activities of the Lichen Secondary Metabolites Vulpinic Acid , (+)-Usnic Acid , and (–)-Usnic Acid against Aerobic and Anaerobic Microorganisms. Antimicrob. Agents Chemother. 39, 2541–2543 (1995). 8585741 10.1128/aac.39.11.2541 PMC162980

[b14] Ingolfsdottir K. Usnic acid. Phytochemistry 61, 729–736 (2002). 12453567 10.1016/s0031-9422(02)00383-7

[b15] Molnár K. & Farkas E. Current results on biological activities of lichen secondary metabolites: a review. Z. Naturforsch. C. 65, 157–73 (2010). 20469633 10.1515/znc-2010-3-401

[b16] Shukla V., Joshi G. P. & Rawat M. S. M. Lichens as a potential natural source of bioactive compounds: a review. Phytochem. Rev. 9, 303–314 (2010).

[b17] Shrestha G. & St. Clair L. L. Lichens: a promising source of antibiotic and anticancer drugs. Phytochem. Rev. 12, 229–244 (2013).

[b18] Bates S. T., Cropsey G. W. G., Caporaso J. G., Knight R. & Fierer N. Bacterial communities associated with the lichen symbiosis. Appl. Environ. Microbiol. 77, 1309–14 (2011). 21169444 10.1128/AEM.02257-10 PMC3067232

[b19] Cardinale M., Berg G., Grube M., Vieira de Castro J. & Müller H. *In situ* analysis of the bacterial community associated with the reindeer lichen Cladonia arbuscula reveals predominance of Alphaproteobacteria. FEMS Microbiol. Ecol. 66, 63–71 (2008). 18631179 10.1111/j.1574-6941.2008.00546.x

[b20] Cardinale M., Puglia A. M. & Grube M. Molecular analysis of lichen-associated bacterial communities. FEMS Microbiol. Ecol. 57, 484–495 (2006). 16907761 10.1111/j.1574-6941.2006.00133.x

[b21] González I., Ayuso-Sacido A., Anderson A. & Genilloud O. Actinomycetes isolated from lichens: evaluation of their diversity and detection of biosynthetic gene sequences. FEMS Microbiol. Ecol. 54, 401–15 (2005). 16332338 10.1016/j.femsec.2005.05.004

[b22] Grube M. & Berg G. Microbial consortia of bacteria and fungi with focus on the lichen symbiosis. Fungal Biol. Rev. 23, 72–85 (2009).

[b23] Grube M., Cardinale M., De Castro J., Mu H. & Berg G. Species-specific structural and functional diversity of bacterial communities in lichen symbioses. Int. Soc. Microb. Ecol. 3, 1105–1115 (2009). 10.1038/ismej.2009.63 19554038

[b24] Hodkinson B. P. & Lutzoni F. A microbiotic survey of lichen-associated bacteria reveals a new lineage from the Rhizobiales. Symbiosis 49, 163–180 (2010).

[b25] Liba C. M. . Nitrogen-fixing chemo-organotrophic bacteria isolated from cyanobacteria-deprived lichens and their ability to solubilize phosphate and to release amino acids and phytohormones. J. Appl. Microbiol. 101, 1076–86 (2006). 17040231 10.1111/j.1365-2672.2006.03010.x

[b26] Muggia L., Klug B., Berg G. & Grube M. Localization of bacteria in lichens from Alpine soil crusts by fluorescence *in situ* hybridization. Appl. Soil Ecol. 68, 20–25 (2013).

[b27] Cardinale M., Grube M., Castro J. V., Müller H. & Berg G. Bacterial taxa associated with the lung lichen Lobaria pulmonaria are differentially shaped by geography and habitat. FEMS Microbiol. Lett. 329, 111–5 (2012). 22268428 10.1111/j.1574-6968.2012.02508.x

[b28] Bjelland T. . Microbial metacommunities in the lichen-rock habitat. Environ. Microbiol. Rep. 3, 434–442 (2011). 23761305 10.1111/j.1758-2229.2010.00206.x

[b29] Sigurbjörnsdóttir M. A., Heiðmarsson S., Jónsdóttir A. R. & Vilhelmsson O. Novel bacteria associated with Arctic seashore lichens have potential roles in nutrient scavenging. Can. J. Microbiol. 92, 307–317 (2014). 24802938 10.1139/cjm-2013-0888

[b30] Kim O.-S. . Introducing EzTaxon-e: a prokaryotic 16S rRNA gene sequence database with phylotypes that represent uncultured species. Int J Syst Evol Microbiol 65, 716–721 (2012). 22140171 10.1099/ijs.0.038075-0

[b31] Navarro-Noya Y. E. . Pyrosequencing Analysis of the Bacterial Community in Drinking Water Wells. Microb. Ecol. 66, 19–29 (2013). 23563631 10.1007/s00248-013-0222-3

[b32] Maldonado L. a . Diversity of cultivable actinobacteria in geographically widespread marine sediments. Antonie Van Leeuwenhoek 87, 11–8 (2005). 15726286 10.1007/s10482-004-6525-0

[b33] Fenical W. & Jensen P. R. Developing a new resource for drug discovery: marine actinomycete bacteria. Nat. Chem. Biol. 2, 666–73 (2006). 17108984 10.1038/nchembio841

[b34] Bredholdt H. . Rare actinomycete bacteria from the shallow water sediments of the Trondheim fjord, Norway: isolation, diversity and biological activity. Environ. Microbiol. 9, 2756–64 (2007). 17922759 10.1111/j.1462-2920.2007.01387.x

[b35] Trujillo M. E. . The genus Micromonospora is widespread in legume root nodules: the example of Lupinus angustifolius. ISME J. 4, 1265–81 (2010). 20445637 10.1038/ismej.2010.55

[b36] Kurtböke D. I. Biodiscovery from rare actinomycetes: an eco-taxonomical perspective. Appl. Microbiol. Biotechnol. 93, 1843–52 (2012). 22297430 10.1007/s00253-012-3898-2

[b37] Ai W. . Axinelline A, a new COX-2 inhibitor from Streptomyces axinellae SCSIO02208. Nat. Prod. Res. 1–6 (2014). doi: 10.1080/14786419.2014.891204. 24666327

[b38] Farris M. H. & Steinberg A. D. Mitrecin A, an endolysin-like bacteriolytic enzyme from a newly isolated soil streptomycete. Lett. Appl. Microbiol. 58, 493–502 (2014). 24460923 10.1111/lam.12220 PMC4238840

[b39] Harunari E. . Hyaluromycin, a new hyaluronidase inhibitor of polyketide origin from marine Streptomyces sp. Mar. Drugs 12, 491–507 (2014). 24451191 10.3390/md12010491 PMC3917283

[b40] He X.-X. . Pelopuradazole, a new imidazole derivative alkaloid from the marine bacteria Pelomonas puraquae sp. nov. Nat. Prod. Res. 1–3 (2014). doi: 10.1080/14786419.2014.891591. 24597911

[b41] Kim K. H. . Natalamycin A, an Ansamycin from a Termite-Associated Streptomyces sp. Chem. Sci. (2014). doi: 10.1039/C4SC01136H. PMC4224317 25386334

[b42] Lee J. . Anmindenols A and B, Inducible Nitric Oxide Synthase Inhibitors from a Marine-Derived Streptomyces sp. J. Nat. Prod. (2014). doi: 10.1021/np500285a. 24878306

[b43] Park H. B., Lee J. K., Lee K. R. & Kwon H. C. Angumycinones A and B, two new angucyclic quinones from Streptomyces sp. KMC004 isolated from acidic mine drainage. Tetrahedron Lett. 55, 63–66 (2014).

[b44] Raju R. . Oleamycins A and B: new antibacterial cyclic hexadepsipeptides isolated from a terrestrial Streptomyces sp. J. Antibiot. (Tokyo). (2014). doi: 10.1038/ja.2014.1. 24448627

[b45] Raju R. . Mollemycin A: An Antimalarial and Antibacterial Glyco-hexadepsipeptide-polyketide from an Australian Marine-Derived Streptomyces sp. (CMB-M0244). Org. Lett. 16, 1716–9 (2014). 24611932 10.1021/ol5003913

[b46] Vartak A. . Isolation of a new broad spectrum antifungal polyene from Streptomyces sp. MTCC 5680. Lett. Appl. Microbiol. (2014). doi: 10.1111/lam.12229. 24517845

[b47] Xu D.-B., Ye W.-W., Han Y., Deng Z.-X. & Hong K. Natural Products from Mangrove Actinomycetes. Mar. Drugs 12, 2590–2613 (2014). 24798926 10.3390/md12052590 PMC4052306

[b48] Zhang J. . Juanlimycins A and B, Ansamycin Macrodilactams from Streptomyces sp. Org. Lett. (2014). doi: 10.1021/ol501072t. 24797062

[b49] Takagi M. & Shin-Ya K. New species of actinomycetes do not always produce new compounds with high frequency. J. Antibiot. (Tokyo). 64, 699–701 (2011). 21792206 10.1038/ja.2011.66

[b50] Hertweck C. The biosynthetic logic of polyketide diversity. Angew. Chem. Int. Ed. Engl. 48, 4688–716 (2009). 19514004 10.1002/anie.200806121

[b51] Davies J. . Uncialamycin, A New Enediyne Antibiotic. Org. Lett. 7, 5233–5236 (2005). 16268546 10.1021/ol052081f

[b52] Williams D. E. . Cladoniamides A-G Tryptophan-Derived Alkaloids Produced in Culture by Streptomyces uncialis. Org. Lett. 10, 3501–3504 (2008). 18646774 10.1021/ol801274c

[b53] Motohashi K., Takagi M., Yamamura H., Hayakawa M. & Shin-ya K. A new angucycline and a new butenolide isolated from lichen-derived Streptomyces spp. J. Antibiot. (Tokyo). 63, 545–548 (2010). 20664606 10.1038/ja.2010.94

[b54] Cheenpracha S., Vidor N. B., Yoshida W. Y., Davies J. & Chang L. C. Coumabiocins A-F , Aminocoumarins from an Organic Extract of Streptomyces sp . L-4-4. J. Nat. Prod. 73, 880–884 (2010). 20384319 10.1021/np900843b

[b55] Schroeckh V. . Intimate bacterial-fungal interaction triggers biosynthesis of archetypal polyketides in Aspergillus nidulans. Proc. Natl. Acad. Sci. USA 106, 14558–14563 (2009). 19666480 10.1073/pnas.0901870106 PMC2732885

[b56] Weisburg W. G., Barns S. M., Pelletier D. A. & Lane D. J. 16S ribosomal DNA amplification for phylogenetic study. J. Bacteriol. 173, 697–703 (1991). 1987160 10.1128/jb.173.2.697-703.1991 PMC207061

[b57] Lane D. J. in Nucleic acid techniques in bacterial systematics. (eds. Stackebrandt E. & Goodfellow M.) 115–175 (John Wiley and Sons, 1991).

[b58] Caporaso J. G. . Global patterns of 16S rRNA diversity at a depth of millions of sequences per sample. Proc. Natl. Acad. Sci. USA 108 Suppl, 4516–22 (2010). 20534432 10.1073/pnas.1000080107 PMC3063599

[b59] Altschul S., Gish W. & Miller W. Basic Local Alignment Search Tool. J Mol Biol. 215, 403–410 (1990). 2231712 10.1016/S0022-2836(05)80360-2

[b60] Courtois S. . Recombinant Environmental Libraries Provide Access to Microbial Diversity for Drug Discovery from Natural Products. Appl. Environ. Microbiol. 69, 49–55 (2003). 12513976 10.1128/AEM.69.1.49-55.2003 PMC152451

[b61] Metsä-Ketelä M. . An efficient approach for screening minimal PKS genes from Streptomyces. FEMS Microbiol. Lett. 180, 1–6 (1999). 10547437 10.1111/j.1574-6968.1999.tb08770.x

[b62] Matsuyama H., Kawasaki K., Yumoto I. & Shida O. Microbacterium kitamiense sp. nov., a new polysaccharide-producing bacterium isolated from the wastewater of a sugar-beet factory. Int. J. Syst. Bacteriol. 49, 1353–1357 (1999). 10555312 10.1099/00207713-49-4-1353

[b63] Deetae P., Spinnler H., Bonnarme P. & Helinck S. Growth and aroma contribution of Microbacterium foliorum, Proteus vulgaris and Psychrobacter sp. during ripening in a cheese model medium. Appl. Microbiol. Biotechnol. 82, 169–177 (2009). 19083231 10.1007/s00253-008-1805-7

[b64] Braña A. F., Fiedler H., Nava H. & Blanco G. Two Streptomyces Species Producing Antibiotic, Antitumor, and Anti-Inflammatory Compounds Are Widespread Among Intertidal Macroalgae and Deep-Sea Coral Reef Invertebrates from the Central Cantabrian Sea. Microb. Ecol. 69, 512–524 (2015). 25319239 10.1007/s00248-014-0508-0

[b65] Bultel-Poncé V., Debitus C., Blond A. & Cerceau C. Lutoside: an Acyl-l-(Acyi-6’.Mannobiosyi)-3-Giycerol Isolated from the Sponge-associated Bacterium Micrococcus luteus. Science. 38, 5805–5808 (1997).

[b66] Barazani O. & Friedman J. Is IAA the major root growth factor secreted from plant-growth-mediating bacteria? J. Chem. Ecol. 25, 2397–2406 (1999).

[b67] Cho J. . Isolation and Structural Determination of the Antifouling Diketopiperazines from Marine-Derived Streptomyces praecox 291-11. Biosci. Biotechnol. Biochem. 76, 1116–1121 (2012). 22790932 10.1271/bbb.110943

[b68] Iwamoto T. . FR109615, A new antifungal antibiotic from Streptomyces setonii: taxonomy, fermentation, isolation, physico-chemical properties and biological activity. J. antiobics 43, 1–7 (1990). 10.7164/antibiotics.43.1 2307620

[b69] Larsen S. H., Boeck L. V. D., Mertz F. P., PaschaL J. W. & Occolowitz J. L. 16-Deethylindanomycin (A83094A), a novel pyrrole-ether antibiotic produced by a strain of Streptomyces setonii. Taxonomy, fermentation, isolation and characterization. J. antiobics 41, 1170–1177 (1988). 10.7164/antibiotics.41.1170 3182398

[b70] Arsen S. H., Berry D. M., Paschal J. W. & Gilliam J. M. 5-Hydroxymethylblasticidin S and blasticidin S from Streptomyces setonii culture A83094. J. antiobics 42, 470–471 (1989). 10.7164/antibiotics.42.470 2708141

[b71] Gurtler H. . Albaflavenone, a sesquiterpene ketone with a zizaene skeleton produced by a streptomycete with a new rope morphology. J. Antibiot. (Tokyo). 47, 434–439 (1994). 8195043 10.7164/antibiotics.47.434

[b72] Yan L. . Antimycin A 18 produced by an endophytic Streptomyces albidoflavus isolated from a mangrove plant. J. Antibiot. (Tokyo). 63, 259–261 (2010). 20300130 10.1038/ja.2010.21

[b73] Roy R. N. & Sen S. K. Fermentation Studies for the Production of Dibutyl Phthalate , an Ester Bioactive Compound from Streptomyces albidoflavus. Jordan J. Biol. Sci. 6, 177–181 (2013).

[b74] Lapchinskaia O., Saburova T., Siniagina O., Konstantinova N. & Filicheva V. Spontaneous and induced variability in Actinomyces rubiginosohelvolus, a new producer of the antibiotic rubomycin. Antibiotiki 20, 1061–1065 (1975). 1225178

[b75] Qi-Lei C., Zi-Han Z. & Lu W. Bromoxantholipin, a novel polycyclic xanthone antibiotic produced by Streptomyces flavogriseus SIIA-A02191. Chinese J. Antibiotcs 36, 566–570 (2011).

[b76] Álvarez-Álvarez R. . Expression of the endogenous and heterologous clavulanic acid cluster in Streptomyces flavogriseus: Why a silent cluster is sleeping. Appl. Microbiol. Biotechnol. 97, 9451–9463 (2013). 23974366 10.1007/s00253-013-5148-7

[b77] Pettit G. R. . Antineoplastic agents. Part 409: Isolation and structure of montanastatin from a terrestrial actinomycete. Bioorganic Med. Chem. 7, 895–899 (1999). 10.1016/s0968-0896(99)00024-3 10400343

[b78] Shinya K., Tauchi T., Morohoshi T. & Ono T. inventors; Sosei Co., Ltd., assignee. GM-95-containing antitumor effect potentiator, combined antitumor preparation and antitumor agent. United States patent US 7,470,714 B2. 2008 Dec 30.

[b79] Gebhardt K. . Endophenazines A-D, new phenazine antibiotics from the Arthropod associated endosymbiont Streptomyces anulatus. J. Antibiot. (Tokyo). 55, 795–800 (2002). 10.7164/antibiotics.55.794 12458768

[b80] Usuki H. . TMG-chitotriomycin, an enzyme inhibitor specific for insect and fungal??-N-acetylglucosaminidases, produced by actinomycete Streptomyces anulatus NBRC 13369. J. Am. Chem. Soc. 130, 4146–4152 (2008). 18307344 10.1021/ja077641f

[b81] Sun D. . A new glutarimide derivative from marine sponge-derived Streptomyces anulatus S71. Nat. Prod. Res. 28, 1602–1606 (2014). 24949797 10.1080/14786419.2014.928877

[b82] Holmalahti J. . Production of dihydroabikoviromycin by Streptomyces anulatus: production parameters and chemical characterization of genotoxicity. J. Appl. Microbiol. 85, 61–68 (1998).

[b83] Tian X. X. . Isolation and identification of poly-α-(1 → 4)-linked 3-O-methyl-D-mannopyranose from a hot-water extract of Mycobacterium vaccae. Carbohydr. Res. 324, 38–44 (2000). 10723610 10.1016/s0008-6215(99)00248-7

[b84] Izumikawa M., Kawahara T., Hwang J., Takagi M. & Shin-Ya K. JBIR-107, a New Metabolite from the Marine-Sponge-Derived Actinomycete, Streptomyces tateyamensis NBRC 105047. Biosci. Biotechnol. Biochem. 77, 663–665 (2013). 23470743 10.1271/bbb.120832

[b85] Häberli A., Bircher C. & Pfander H. Isolation of a new carotenoid and two new carotenoid glycosides from Curtobacterium flaccumfaciens pvar poinsettiae. Helv. Chim. Acta 83, 328–335 (2000).

[b86] Osawa A. . Characterization and antioxidative activities of rare C(50) carotenoids-sarcinaxanthin, sarcinaxanthin monoglucoside, and sarcinaxanthin diglucoside-obtained from Micrococcus yunnanensis. J. Oleo Sci. 59, 653–659 (2010). 21099143 10.5650/jos.59.653

[b87] Zhou X. . New anti-infective cycloheptadepsipeptide congeners and absolute stereochemistry from the deep sea-derived Streptomyces drozdowiczii SCSIO 10141. Tetrahedron 70, 7795–7801 (2014).

[b88] Hara M. . Leinamycin, a new antitumor antibiotic from Streptomyces: producing organism, fermentation and isolation. J. Antibiot. (Tokyo). 42, 1768–1774 (1989). 2621160 10.7164/antibiotics.42.1768

[b89] Kodai S. & Ninomiya A. Isolation of New Thiopeptide Berninamycin E from Streptomyces atroolivaceus. Asian J. Chem. 25, 490–492 (2013).

[b90] Shashkov A., Tul’skaya E., Evtushenko L. & Naumova I. A teichoic acid of Nocardioides albus VKM Ac-805(T) cell walls. Biochemistry 64, 1305–1309 (1999). 10611537

[b91] Dellweg H. . Rodaplutin, a new peptidylnucleoside from Nocardioides albus. J. Antibiot. (Tokyo). 41, 1145–1147 (1988). 3170347 10.7164/antibiotics.41.1145

