# Supplementary material for: Littoral lichens as a novel source of potentially bioactive Actinobacteria
Source: Sci Rep. 2015 Oct 30;5:15839. doi: 10.1038/srep15839 (PMC4626775; doi:10.1038/srep15839)
Supplement: Supplementary Information [file srep15839-s1.doc]

**Supplementary materials**

**Littoral lichens as a novel source of potentially bioactive Actinobacteria**

Delphine PARROT^1^, Sanjay ANTONY-BABU^2,3^, Laurent INTERTAGLIA^2,4^, Martin GRUBE^5^, Sophie TOMASI^1^, Marcelino T. SUZUKI^2,3*^

^1^*UMR CNRS 6226, Institut des Sciences chimiques de Rennes, Equipe PNSCM “Produits Naturels – Synthèses – Chimie Médicinale”, UFR Sciences Pharmaceutiques et Biologiques, Univ. Rennes 1, Université Européenne de Bretagne, 2 Avenue du Pr. Léon Bernard, F-35043 Rennes, France*

^2^*Sorbonne Universités; UPMC Univ. Paris VI, UMS 2348, USR 3579 LBBM, Observatoire Océanologique, Banyuls-sur-Mer 66650, France*

^3^*CNRS, USR 3579, LBBM, Observatoire Océanologique, F-66650, Banyuls/Mer, France*

^4^*CNRS, UMS 2348 (Plate-forme Bio2Mar), Observatoire Océanologique, F-66650 Banyuls/Mer, France*

^5^*Institut für Pflanzenwissenschaften Karl-Franzens-Universität Graz, Austria*

^*^ Corresponding author. Tel.: +33 (0) 430192401

*E-mail address:* [suzuki@obs-banyuls.fr](mailto:suzuki@obs-banyuls.fr) (M.T. Suzuki)

**Table S1:** List of the cultivable bacteria isolated from the four lichens according on the culture conditions: media (MA: Marine Agar media. AIA: Actinomycetes Isolation Agar media and ISP2: International Streptomyces Project 2 Agar media) and bacterial extract (Wash and Homogenate extract), length is bp for the 16S rRNA sequence, closest relative based on EZtaxon searches, percentage similarities to closest relative and phylogenetic identification of closest relative

| **Strain** | ***Lichen*** | **Environment** | **Media** | **Extracts** | **Length (bp)** | **Closest relative in Eztaxon server** | **Accession number (Eztaxon)** | **% 16S^*^** | **Phylum/Class** | **Family** |
| --- | --- | --- | --- | --- | --- | --- | --- | --- | --- | --- |
| **MOLA1501** | *Lichina confinis* | Marine | MA | Homogenate | 802 | *Sphingorhabdus wooponensis* | HQ436493 | **95.74** | *Alphaproteobacteria* | *Sphingomonadaceae* |
| **MOLA1487** | *Lichina confinis* | Marine | AIA | Wash | 778 | *Saccharopolyspora gloriosae* | EU005371 | 100.00 | *Actinobacteria* | *Pseudonocardiaceae* |
| **MOLA1509** | *Lichina confinis* | Marine | AIA | Wash | 838 |  |  |  |  |  |
| **MOLA1499** | *Lichina confinis* | Marine | MA | Wash | 791 | *Jannaschia faecimaris* | JX680802 | 97.47 | *Alphaproteobacteria* | *Rhodobacteraceae* |
| **MOLA1443** | *Lichina confinis* | Marine | MA | Homogenate | 800 |  |  |  |  |  |
| **MOLA1508** | *Lichina confinis* | Marine | AIA | Homogenate | 844 | *Microbacterium aurantiacum* | EU863415 | 98.71 | *Actinobacteria* | *Microbacteriaceae* |
| **MOLA1503** | *Lichina confinis* | Marine | AIA | Homogenate | 844 |  |  |  |  |  |
| **MOLA1515** | *Lichina confinis* | Marine | AIA | Wash | 848 | *Agromyces terreus* | EF363711 | 97.26 | *Actinobacteria* | *Microbacteriaceae* |
| **MOLA1514** | *Lichina confinis* | Marine | AIA | Wash | 795 | *Rhizorhapis suberifaciens* | KF437561 | **95.39** | *Alphaproteobacteria* | *Sphingomonadaceae* |
| **MOLA1512** | *Lichina confinis* | Marine | AIA | Homogenate | 840 | *Sediminihabitans luteus* | AB695376 | 99.28 | *Actinobacteria* | *Sanguibacteraceae* |
| **MOLA1486** | *Lichina confinis* | Marine | AIA | Wash | 829 | *Microbacterium foliorum* | AJ249780 | 99.39 | *Actinobacteria* | *Microbacteriaceae* |
| **MOLA1466** | *Lichina confinis* | Marine | MA | Wash | 786 | *Sulfitobacter marinus* | DQ683726 | 99.22 | *Alphaproteobacteria* | *Rhodobacteraceae* |
| **MOLA1498** | *Lichina confinis* | Marine | MA | Wash | 799 | *Profundibacterium mesophilum* | JF776971 | **96.06** | *Alphaproteobacteria* | *Rhodobacteraceae* |
| **MOLA1497** | *Lichina confinis* | Marine | MA | Wash | 833 | *Lewinella persica* | ARDG01000103 | **94.36** | *Alphaproteobacteria* | *Saprospiraceae* |
| **MOLA1495** | *Lichina confinis* | Marine | MA | Wash | 809 | *Sulfitobacter brevis* | Y16425 | 98.85 | *Alphaproteobacteria* | *Rhodobacteraceae* |
| **MOLA1494** | *Lichina confinis* | Marine | MA | Wash | 809 |  |  |  |  |  |
| **MOLA1488** | *Lichina confinis* | Marine | AIA | Wash | 826 | *Streptomyces cyaneofuscatus* | AY999770 | 100.00 | *Actinobacteria* | *Streptomycetaceae* |
| **MOLA1493** | *Lichina confinis* | Marine | AIA | Wash | 806 |  |  |  |  |  |
| **MOLA1492** | *Lichina confinis* | Marine | AIA | Wash | 824 | *Streptomyces carpaticus* | AB184641 | 99.75 | *Actinobacteria* | *Streptomycetaceae* |
| **MOLA1491** | *Lichina confinis* | Marine | AIA | Wash | 832 | *Kocuria rhizophila* | Y16264 | 99.51 | *Actinobacteria* | *Micrococcaceae* |
| **MOLA1490** | *Lichina confinis* | Marine | AIA | Wash | 819 | *Micrococcus luteus* | CP001628 | 98.90 | *Actinobacteria* | *Micrococcaceae* |
| **MOLA1489** | *Lichina confinis* | Marine | AIA | Wash | 672 | *Tersicoccus phoenicis* | EU977596 | 98.96 | *Actinobacteria* | *Micrococcaceae* |
| **MOLA1485** | *Lichina confinis* | Marine | AIA | Wash | 828 | *Bacillus simplex* | AB363738 | 100.00 | *Firmicutes* | *Bacillaceae* |
| **MOLA1484** | *Lichina confinis* | Marine | AIA | Homogenate | 812 | *Streptomyces setonii* | AB184300 | 100.00 | *Actinobacteria* | *Streptomycetaceae* |
| **MOLA1464** | *Lichina confinis* | Marine | MA | Wash | 805 | *Rhizorhapis suberifaciens* |  | **96.15** | *Alphaproteobacteria* | *Sphingomonadaceae* |
| **MOLA1465** | *Lichina confinis* | Marine | MA | Wash | 805 |  | KF437561 |  |  |  |
| **MOLA1463** | *Lichina confinis* | Marine | MA | Wash | 805 |  |  |  |  |  |
| **MOLA1457** | *Lichina confinis* | Marine | MA | Wash | 795 | *Sulfitobacter brevis* | Y16425 | 99.09 | *Alphaproteobacteria* | *Rhodobacteraceae* |
| **MOLA1458** | *Lichina confinis* | Marine | MA | Wash | 795 |  |  |  |  |  |

| **MOLA1461** | | *Lichina confinis* | Marine | MA | Wash | 793 | *Erythrobacter citreus* |  | 98.59 | *Alphaproteobacteria* | *Erythrobacteraceae* |
| --- | --- | --- | --- | --- | --- | --- | --- | --- | --- | --- | --- |
| **MOLA1462** | | *Lichina confinis* | Marine | MA | Wash | 793 |  | AF118020 |  |  |  |
| **MOLA1460** | | *Lichina confinis* | Marine | MA | Wash | 793 |  |  |  |  |  |
| **MOLA 1449** | | *Lichina confinis* | Marine | MA | Homogenate | 793 |  |  |  |  |  |
| **MOLA1456** | | *Lichina confinis* | Marine | MA | Wash | 620 | *Porphyrobacter sanguineus* | AB021493 | 97.09 | *Alphaproteobacteria* | *Erythrobacteraceae* |
| **MOLA1451** | | *Lichina confinis* | Marine | MA | Homogenate | 793 | *Parasphingopyxis lamellibrachiae* | AB524074 | **93.57** | *Alphaproteobacteria* | *Rhodobacteraceae* |
| **MOLA1452** | | *Lichina confinis* | Marine | MA | Wash | 793 |  |  |  |  |  |
| **MOLA1453** | | *Lichina confinis* | Marine | MA | Wash | 793 |  |  |  |  |  |
| **MOLA1454** | | *Lichina confinis* | Marine | MA | Wash | 793 |  |  |  |  |  |
| **MOLA1447** | | *Lichina confinis* | Marine | MA | Homogenate | 823 | *Paraoerskovia sediminicola* | AB695378 | 99.03 | *Actinobacteria* | *Cellulomonadaceae* |
| **MOLA1439** | | *Lichina confinis* | Marine | MA | Homogenate | 793 | *Paracoccus carotinifaciens* | AB006899 | 98.61 | *Alphaproteobacteria* | *Paracoccaceae* |
| **MOLA1437** | | *Lichina confinis* | Marine | MA | Homogenate | 793 |  |  |  |  |  |
| **MOLA1500** | | *Lichina confinis* | Marine | MA | Homogenate | 793 |  |  |  |  |  |
| **MOLA1438** | | *Lichina confinis* | Marine | MA | Homogenate | 793 |  |  |  |  |  |
| **MOLA1450** | | *Lichina confinis* | Marine | MA | Homogenate | 831 | *Cellulosimicrobium terreum* | EF076760 | 97.93 | *Actinobacteria* | *Promicromonosporaceae* |
| **MOLA1448** | | *Lichina confinis* | Marine | MA | Homogenate | 839 | *Nocardioides mesophilus* | EF466117 | **95.04** | *Actinobacteria* | *Nocardioidaceae* |
|  | |  |  |  |  |  |  | AM295338 |  |  |  |
| **MOLA1446** | | *Lichina confinis* | Marine | MA | Homogenate | 849 | *Marmoricola aequoreus* |  | 98.40 | *Actinobacteria* | *Nocardioidaceae* |
| **MOLA1445** | | *Lichina confinis* | Marine | MA | Homogenate | 838 | *Micrococcus aloeverae* | KF524364 | 100 | *Actinobacteria* | *Micrococcaceae* |
| **MOLA1442** | | *Lichina confinis* | Marine | MA | Homogenate | 794 | *Tropicibacter litoreus* | HE860713 | **96.21** | *Alphaproteobacteria* | *Rhodobacteraceae* |
| **MOLA1502** | | *Lichina confinis* | Marine | MA | Homogenate | 796 |  |  |  |  |  |
| **MOLA1441** | | *Lichina confinis* | Marine | MA | Homogenate | 847 | *Agromyces terreus* | EF363711 | **96.04** | *Actinobacteria* | *Microbacteriaceae* |
| **MOLA1513** | | *Lichina confinis* | Marine | AIA | Homogenate | 847 |  |  |  |  |  |
| **MOLA1440** | | *Lichina confinis* | Marine | MA | Homogenate | 804 | *Tropicibacter litoreus* | HE860713 | **95.58** | *Alphaproteobacteria* | *Rhodobacteraceae* |
| **MOLA1455** | | *Lichina confinis* | Marine | MA | Wash | 798 | *Pacificamonas flava* | JX845636 | **95.87** | *Alphaproteobacteria* | *Sphingomonadaceae* |
| **MOLA1496** | | *Lichina confinis* | Marine | MA | Wash | 818 | *Sphingomonas adhaesiva* | D13722 | **96.02** | *Alphaproteobacteria* | *Sphingomonadaceae* |
| **MOLA1444** | | *Lichina confinis* | Marine | MA | Homogenate | 842 | *Brevibacterium epidermidis* | X76565 | 98.42 | *Actinobacteria* | *Brevibacteriaceae* |
| **MOLA1436** | | *Lichina pygmaea* | Marine | MA | Wash | 797 | *Sulfitobacter brevis Ekho* | Y16425 | 98.86 | *Alphaproteobacteria* | *Rhodobacteraceae* |
| **MOLA1434** | | *Lichina pygmaea* | Marine | MA | Wash | 833 | *Marmoricola aequoreus* | AM295338 | 98.39 | *Actinobacteria* | *Nocardioidaceae* |
| **MOLA1433** | | *Lichina pygmaea* | Marine | MA | Homogenate | 793 |  |  |  |  |  |
| **MOLA1411** | | *Lichina pygmaea* | Marine | MA | Wash | 793 | *Loktanella hongkongensis* | APGJ01000007 | 99.62 | *Alphaproteobacteria* | *Rhodobacteraceae* |
| **MOLA1435** | | *Lichina pygmaea* | Marine | MA | Wash | 836 | *Brevibacterium picturae* | AJ620364 | 99.52 | *Actinobacteria* | *Brevibacteriaceae* |
| **MOLA1432** | | *Lichina pygmaea* | Marine | MA | Homogenate | 867 | *Staphylococcus epidermidis* | L37605 | 100.00 | *Firmicutes* | *Staphylococcaceae* |
|  |  | | | | | | | | | | |

| **MOLA1510** | *Lichina pygmaea* | Marine | MA | Homogenate | 802 | *Hoeflea phototrophica* |  | **91.90** | *Alphaproteobacteria* | *Phyllobacteriaceae* |
| --- | --- | --- | --- | --- | --- | --- | --- | --- | --- | --- |
| **MOLA1416** | *Lichina pygmaea* | Marine | MA | Homogenate | 802 |  |  |  |  |  |
| **MOLA1507** | *Lichina pygmaea* | Marine | MA | Homogenate | 780 |  |  |  |  |  |
| **MOLA1504** | *Lichina pygmaea* | Marine | MA | Homogenate | 802 |  | ABIA02000018 |  |  |  |
| **MOLA1505** | *Lichina pygmaea* | Marine | MA | Homogenate | 802 |  |  |  |  |  |
| **MOLA1415** | *Lichina pygmaea* | Marine | MA | Homogenate | 802 |  |  |  |  |  |
| **MOLA1511** | *Lichina pygmaea* | Marine | MA | Homogenate | 802 |  |  |  |  |  |
| **MOLA1427** | *Lichina pygmaea* | Marine | AIA | Homogenate | 829 | *Streptomyces cyaneofuscatus* | AY999770 | 100.00 | *Actinobacteria* | *Streptomycetaceae* |
| **MOLA1426** | *Lichina pygmaea* | Marine | AIA | Homogenate | 865 | *Bacillus tequilensis* | AYTO01000043 | 99.77 | *Firmicutes* | *Bacillaceae* |
| **MOLA1425** | *Lichina pygmaea* | Marine | MA | Homogenate | 829 | *Streptomyces albidoflavus* | Z76676 | 100.00 | *Actinobacteria* | *Streptomycetaceae* |
| **MOLA1405** | *Lichina pygmaea* | Marine | AIA | Homogenate | 829 |  |  |  |  |  |
| **MOLA1424** | *Lichina pygmaea* | Marine | MA | Homogenate | 866 | *Bacillus aryabhattai* | EF114313 | 99.65 | *Firmicutes* | *Bacillaceae* |
| **MOLA1423** | *Lichina pygmaea* | Marine | MA | Homogenate | 695 | *Bacillus licheniformis* | AE017333 | 99.71 | *Firmicutes* | *Bacillaceae* |
| **MOLA1422** | *Lichina pygmaea* | Marine | MA | Homogenate | 695 |  |  |  |  |  |
| **MOLA1421** | *Lichina pygmaea* | Marine | MA | Homogenate | 815 | *Streptomyces cyaneofuscatus* | AY999770 | 99.88 | *Actinobacteria* | *Streptomycetaceae* |
| **MOLA1420** | *Lichina pygmaea* | Marine | MA | Homogenate | 815 |  |  |  |  |  |
| **MOLA1419** | *Lichina pygmaea* | Marine | MA | Homogenate | 825 | *Halobacillus locisalis* | AY190534 | 99.64 | *Firmicutes* | *Bacillaceae* |
| **MOLA1418** | *Lichina pygmaea* | Marine | MA | Homogenate | 814 | *Micrococcus aloeverae* | KF524364 | 100.00 | *Actinobacteria* | *Micrococcaceae* |
| **MOLA1417** | *Lichina pygmaea* | Marine | MA | Homogenate | 777 | *Erythrobacter citreus* | AF118020 | 98.84 | *Alphaproteobacteria* | *Erythrobacteraceae* |
| **MOLA1409** | *Lichina pygmaea* | Marine | MA | Wash | 860 | *Bacillus hwajinpoensis* | AF541966 | 99.42 | *Firmicutes* | *Bacillaceae* |
| **MOLA1410** | *Lichina pygmaea* | Marine | MA | Wash | 860 |  |  |  |  |  |
| **MOLA1412** | *Lichina pygmaea* | Marine | MA | Wash | 860 |  |  |  |  |  |
| **MOLA1408** | *Lichina pygmaea* | Marine | AIA | Homogenate | 824 | *Streptomyces rubiginosohelvolus* | AB184240 | 100.00 | *Actinobacteria* | *Streptomycetaceae* |
| **MOLA1407** | *Lichina pygmaea* | Marine | AIA | Homogenate | 831 | *Salinibacterium amurskyense* | AF539697 | 100.00 | *Actinobacteria* | *Microbacteriaceae* |
| **MOLA1406** | *Lichina pygmaea* | Marine | AIA | Homogenate | 824 | *Streptomyces anulatus* | DQ026637 | 100.00 | *Actinobacteria* | *Streptomycetaceae* |
| **MOLA1431** | *Lichina pygmaea* | Marine | MA | Homogenate | 798 | *Erythrobacter citreus* | AF118020 | 98.10 | *Alphaproteobacteria* | *Erythrobacteraceae* |
| **MOLA1429** | *Lichina pygmaea* | Marine | MA | Homogenate | 801 | *Altererythrobacter namhicola* | FJ935793 | 98.63 | *Alphaproteobacteria* | *Erythrobacteraceae* |
| **MOLA1414** | *Lichina pygmaea* | Marine | MA | Homogenate | 862 | *Planococcus maritimus* | AF500007 | 98.60  **95.83** | *Firmicutes* | *Planococcaceae* |
| **MOLA1413** | *Lichina pygmaea* | Marine | MA | Homogenate | 627 |  |  |  |  |  |
| **MOLA1506** | *Lichina pygmaea* | Marine | MA | Homogenate | 788 | *Altererythrobacter gangjinensis* | JF751048 | 97.44 | *Alphaproteobacteria* | *Erythrobacteraceae* |
| **MOLA1430** | *Lichina pygmaea* | Marine | AIA | Homogenate | 806 | *Altererythrobacter namhicola* | FJ935793 | 98.24 | *Alphaproteobacteria* | *Erythrobacteraceae* |

| **MOLA1551** | | *Roccella fuciformis* | | Maritime | | MA | | Wash | | 801 | | *Phenylobacterium haematophilum* | | | AJ244650 | 98.21 | | *Alphaproteobacteria* | | *Caulobacteraceae* | |
| --- | --- | --- | --- | --- | --- | --- | --- | --- | --- | --- | --- | --- | --- | --- | --- | --- | --- | --- | --- | --- | --- |
| **MOLA1548** | | *Roccella fuciformis* | | Maritime | | MA | | Wash | | 825 | | *Microbacterium murale* | | | HE585693 | 97.27 | | *Actinobacteria* | | *Microbacteriaceae* | |
| **MOLA1545** | | *Roccella fuciformis* | | Maritime | | MA | | Wash | | 829 | | *Microbacterium pumilum* | | | AB234027 | 99.88 | | *Actinobacteria* | | *Microbacteriaceae* | |
| **MOLA1544** | | *Roccella fuciformis* | | Maritime | | MA | | Wash | | 826 | | *Cellulosimicrobium terreum* | | | EF076760 | 97.93 | | *Actinobacteria* | | *Promicromonosporaceae* | |
| **MOLA1542** | | *Roccella fuciformis* | | Maritime | | MA | | Wash | | 803 | | *Paracoccus limosus* | | | HQ336256 | 98.38 | | *Alphaproteobacteria* | | *Paraccocaceae* | |
| **MOLA1535** | | *Roccella fuciformis* | | Maritime | | MA | | Wash | | 803 | |  |  |  |  |  |  |  |  |  |  |
| **MOLA1540** | | *Roccella fuciformis* | | Maritime | | MA | | Wash | | 789 | | *Oceanobacillus profundus* | | | DQ386635 | 99.35 | | *Firmicutes* | | *Bacillaceae* | |
| **MOLA1538** | | *Roccella fuciformis* | | Maritime | | MA | | Wash | | 830 | | *Aeromicrobium tamlense* | | | DQ411541 | 97.75 | | *Actinobacteria* | | *Nocardioidaceae* | |
| **MOLA1536** | | *Roccella fuciformis* | | Maritime | | MA | | Wash | | 826 | | *Sediminihabitans luteus* | | | AB695376 | 98.77 | | *Actinobacteria* | | *Sanguibacteraceae* | |
|  | |  | |  | |  | |  | |  | |  | | | AB695376 |  | |  | |  | |
| **MOLA1534** | | *Roccella fuciformis* | | Maritime | | MA | | Wash | | 829 | | *Sediminihabitans luteus* | | |  | 99.51 | | *Actinobacteria* | | *Sanguibacteraceae* | |
| **MOLA1533** | | *Roccella fuciformis* | | Maritime | | MA | | Wash | | 829 | |  |  |  |  |  |  |  |  |  |  |
| **MOLA1530** | | *Roccella fuciformis* | | Maritime | | MA | | Wash | | 827 | | *Mycobacterium vanbaalenii* | | | CP000511 | 99.15 | | *Actinobacteria* | | *Mycobacteriaceae* | |
| **MOLA1529** | | *Roccella fuciformis* | | Maritime | | MA | | Wash | | 792 | | *Mesorhizobium qingshengii* | | | JQ339788 | 98.10 | | *Alphaproteobacteria* | | *Phyllobacteriaceae* | |
| **MOLA1528** | | *Roccella fuciformis* | | Maritime | | MA | | Wash | | 830 | | *Sediminihabitans luteus* | | | AB695376 | 99.64 | | *Actinobacteria* | | *Sanguibacteraceae* | |
| **MOLA1524** | | *Roccella fuciformis* | | Maritime | | MA | | Wash | | 831 | | *Microbacterium invictum* | | | AM949677 | 98.19 | | *Actinobacteria* | | *Microbacteriaceae* | |
| **MOLA1525** | | *Roccella fuciformis* | | Maritime | | MA | | Wash | | 831 | |  |  |  |  |  |  |  |  |  |  |
| **MOLA1522** | | *Roccella fuciformis* | | Maritime | | MA | | Wash | | 828 | | *Nocardioides mesophilus* | | | EF466117 | **94.87** | | *Actinobacteria* | | *Nocardioidaceae* | |
| **MOLA1523** | | *Roccella fuciformis* | | Maritime | | MA | | Wash | | 828 | |  |  |  |  |  |  |  |  |  |  |
| **MOLA1521** | | *Roccella fuciformis* | | Maritime | | MA | | Wash | | 831 | | *Nocardioides mesophilus* | | |  | **94.86** | | *Actinobacteria* | | *Nocardioidaceae* | |
| **MOLA1599** | | *Roccella fuciformis* | | Maritime | | MA | | Homogenate | | 831 | |  |  |  | EF466117 |  |  |  |  |  |  |
| **MOLA1553** | | *Roccella fuciformis* | | Maritime | | MA | | Homogenate | | 836 | | *Cellulosimicrobium terreum* | | | EF076760 | 97.82 | | *Actinobacteria* | | *Promicromonosporaceae* | |
| **MOLA1520** | | *Roccella fuciformis* | | Maritime | | MA | | Wash | | 836 | |  |  |  |  |  |  |  |  |  |  |
| **MOLA1519** | | *Roccella fuciformis* | | Maritime | | MA | | Wash | | 836 | |  |  |  |  |  |  |  |  |  |  |
| **MOLA1561** | | *Roccella fuciformis* | | Maritime | | MA | | Homogenate | | 836 | |  |  |  |  |  |  |  |  |  |  |
| **MOLA1572** | | *Roccella fuciformis* | | Maritime | | MA | | Homogenate | | 836 | |  |  |  |  |  |  |  |  |  |  |
| **MOLA1518** | | *Roccella fuciformis* | | Maritime | | MA | | Wash | | 826 | | *Isoptericola nanjingensis* | | | HQ222356 | 99.00 | | *Actinobacteria* | | *Promicromonosporaceae* | |
| **MOLA1517** | | *Roccella fuciformis* | | Maritime | | MA | | Wash | | 826 | |  |  |  |  |  |  |  |  |  |  |
| **MOLA1516** | | *Roccella fuciformis* | | Maritime | | MA | | Wash | | 822 | | *Streptomyces tateyamensis* | | | AB473555 | 99.76 | | *Actinobacteria* | | *Streptomycetaceae* | |
| **MOLA1597** | | *Roccella fuciformis* | | Maritime | | AIA | | Wash | | 835 | | *Agromyces terreus* | | | EF363711 | 99.27 | | *Actinobacteria* | | *Microbacteriaceae* | |
| **MOLA1593** | | *Roccella fuciformis* | | Maritime | | AIA | | Homogenate | | 771 | | *Gordonia hankookensis* | | | FJ572038 | 97.27 | | *Actinobacteria* | | *Gordoniaceae* | |
| **MOLA1590** | | *Roccella fuciformis* | | Maritime | | AIA | | Homogenate | | 833 | | *Microbacterium paraoxydans* | | | AJ491806 | 99.76 | | *Actinobacteria* | | *Microbacteriaceae* | |
| **MOLA1589** | | *Roccella fuciformis* | | Maritime | | AIA | | Homogenate | | 832 | | *Curtobacterium flaccumfaciens* | | | AJ312209 | 100.00 | | *Actinobacteria* | | *Microbacteriaceae* | |
| **MOLA1588** | *Roccella fuciformis* | | Maritime | | AIA | | Homogenate | | 721 | | *Mycobacterium flavescens* | | | X52932 | | | 98.58 | | *Actinobacteria* | | *Mycobacteriaceae* |
| **MOLA1554** | *Roccella fuciformis* | | Maritime | | MA | | Homogenate | | 838 | | *Sediminihabitans luteus* | | | AB695376 | | | 99.39 | | *Actinobacteria* | | *Sanguibacteraceae* |
| **MOLA1555** | *Roccella fuciformis* | | Maritime | | MA | | Homogenate | | 838 | |  |  |  |  |  |  |  |  |  |  |  |
| **MOLA1526** | *Roccella fuciformis* | | Maritime | | MA | | Wash | | 838 | |  |  |  |  |  |  |  |  |  |  |  |
| **MOLA1527** | *Roccella fuciformis* | | Maritime | | MA | | Wash | | 838 | |  |  |  |  |  |  |  |  |  |  |  |
| **MOLA1585** | *Roccella fuciformis* | | Maritime | | AIA | | Homogenate | | 838 | |  |  |  |  |  |  |  |  |  |  |  |
| **MOLA1592** | *Roccella fuciformis* | | Maritime | | AIA | | Homogenate | | 838 | |  |  |  |  |  |  |  |  |  |  |  |
| **MOLA1595** | *Roccella fuciformis* | | Maritime | | MA | | Wash | | 838 | |  |  |  |  |  |  |  |  |  |  |  |
| **MOLA1602** | *Roccella fuciformis* | | Maritime | | AIA | | Homogenate | | 838 | |  |  |  |  |  |  |  |  |  |  |  |
| **MOLA1584** | *Roccella fuciformis* | | Maritime | | AIA | | Homogenate | | 829 | | *Microbacterium kitamiense* | | | AB013919 | | | 97.71 | | *Actinobacteria* | | *Microbacteriaceae* |
| **MOLA1594** | *Roccella fuciformis* | | Maritime | | AIA | | Homogenate | | 829 | |  |  |  |  |  |  |  |  |  |  |  |
| **MOLA1583** | *Roccella fuciformis* | | Maritime | | AIA | | Homogenate | | 844 | | *Gordonia hankookensis* | | | FJ572038 | | | 99.88 | | *Actinobacteria* | | *Gordoniaceae* |
| **MOLA1591** | *Roccella fuciformis* | | Maritime | | AIA | | Homogenate | | 844 | |  |  |  |  |  |  |  |  |  |  |  |
| **MOLA1587** | *Roccella fuciformis* | | Maritime | | AIA | | Homogenate | | 844 | |  |  |  |  |  |  |  |  |  |  |  |
| **MOLA1586** | *Roccella fuciformis* | | Maritime | | AIA | | Homogenate | | 844 | |  |  |  |  |  |  |  |  |  |  |  |
| **MOLA1582** | *Roccella fuciformis* | | Maritime | | AIA | | Homogenate | | 823 | | *Gordonia defluvii* | | | AY650265 | | | 98.42 | | *Actinobacteria* | | *Gordoniaceae* |
| **MOLA1531** | *Roccella fuciformis* | | Maritime | | MA | | Wash | | 823 | |  |  |  |  |  |  |  |  |  |  |  |
| **MOLA1580** | *Roccella fuciformis* | | Maritime | | AIA | | Homogenate | | 831 | | *Isoptericola nanjingensis* | | | HQ222356 | | | 99.63 | | *Actinobacteria* | | *Promicromonosporaceae* |
| **MOLA1579** | *Roccella fuciformis* | | Maritime | | AIA | | Homogenate | | 819 | | *Streptomyces brevispora* | | | FR692104 | | | 99.76 | | *Actinobacteria* | | *Streptomycetaceae* |
| **MOLA1578** | *Roccella fuciformis* | | Maritime | | AIA | | Homogenate | | 839 | | *Streptomyces cyaneofuscatus* | | | AY999770 | | | 100.00 | | *Actinobacteria* | | *Streptomycetaceae* |
| **MOLA1596** | *Roccella fuciformis* | | Maritime | | AIA | | Wash | | 839 | |  |  |  |  |  |  |  |  |  |  |  |
| **MOLA1611** | *Roccella fuciformis* | | Maritime | | AIA | | Wash | | 839 | |  |  |  |  |  |  |  |  |  |  |  |
| **MOLA1612** | *Roccella fuciformis* | | Maritime | | AIA | | Wash | | 839 | |  |  |  |  |  |  |  |  |  |  |  |
| **MOLA1615** | *Roccella fuciformis* | | Maritime | | AIA | | Wash | | 839 | |  |  |  |  |  |  |  |  |  |  |  |
| **MOLA1614** | *Roccella fuciformis* | | Maritime | | AIA | | Wash | | 839 | |  |  |  |  |  |  |  |  |  |  |  |
| **MOLA1577** | *Roccella fuciformis* | | Maritime | | MA | | Wash | | 833 | | *Staphylococcus epidermidis* | | | L37605 | | | 100.00 | | *Firmicutes* | | *Staphylococcaceae* |
| **MOLA1576** | *Roccella fuciformis* | | Maritime | | MA | | Wash | | 830 | | *Paraoerskovia marina* | | | JNIY01000001 | | | 98.31 | | *Actinobacteria* | | *Cellulomonadaceae* |
| **MOLA1575** | *Roccella fuciformis* | | Maritime | | MA | | Homogenate | | 841 | | *Micrococcus yunnanensis* | | | FJ214355 | | | 99.75 | | *Actinobacteria* | | *Micrococcaceae* |
| **MOLA1608** | *Roccella fuciformis* | | Maritime | | MA | | Wash | | 841 | |  |  |  |  |  |  |  |  |  |  |  |
| **MOLA1574** | *Roccella fuciformis* | | Maritime | | MA | | Homogenate | | 829 | | *Sediminihabitans luteus* | | | AB695376 | | | 99.76 | | *Actinobacteria* | | *Sanguibacteraceae* |
| **MOLA1573** | *Roccella fuciformis* | | Maritime | | MA | | Homogenate | | 819 | | *Cellulosimicrobium terreum* | | | EF076760 | | | 97.80 | | *Actinobacteria* | | *Promicromonosporaceae* |
| **MOLA1570** | *Roccella fuciformis* | | Maritime | | MA | | Homogenate | | 847 | | *Agromyces terreus* | | | EF363711 | | | 97.58 | | *Actinobacteria* | | *Microbacteriaceae* |
| **MOLA1569** | *Roccella fuciformis* | | Maritime | | MA | | Homogenate | | 834 | | *Brevibacterium epidermidis* | | | X76565 | | | 98.53 | | *Actinobacteria* | | *Brevibacteriaceae* |
|  |  |  |  |  |  |  |  |  | 834 | |  | |  | | | | | | | | |

| **MOLA1567** | *Roccella fuciformis* | Maritime | MA | Homogenate | 829 | *Microbacterium oleivorans* | AJ698725 | 98.31 | *Actinobacteria* | *Microbacteriaceae* |
| --- | --- | --- | --- | --- | --- | --- | --- | --- | --- | --- |
| **MOLA1566** | *Roccella fuciformis* | Maritime | MA | Homogenate | 829 |
| **MOLA1564** | *Roccella fuciformis* | Maritime | MA | Homogenate | 829 |
| **MOLA1565** | *Roccella fuciformis* | Maritime | MA | Homogenate | 821 | *Brevibacterium epidermidis* | X76565 | 98.40 | *Actinobacteria* | *Brevibacteriaceae* |
| **MOLA1563** | *Roccella fuciformis* | Maritime | MA | Homogenate | 839 | *Sediminihabitans luteus* | AB695376 | 98.07 | *Actinobacteria* | *Sanguibacteraceae* |
| **MOLA1549** | *Roccella fuciformis* | Maritime | MA | Wash | 839 |
| **MOLA1537** | *Roccella fuciformis* | Maritime | MA | Wash | 839 |
| **MOLA1562** | *Roccella fuciformis* | Maritime | MA | Homogenate | 829 | *Microbacterium suwononse* | GQ246683 | 98.31 | *Actinobacteria* | *Microbacteriaceae* |
| **MOLA1560** | *Roccella fuciformis* | Maritime | MA | Homogenate | 838 | *Paraoerskovia sediminicola* | AB695378 | 98.56 | *Actinobacteria* | *Cellulomonadaceae* |
| **MOLA1559** | *Roccella fuciformis* | Maritime | MA | Homogenate | 830 | *Paraoerskovia sediminicola* | AB695378 | 99.03 | *Actinobacteria* | *Cellulomonadaceae* |
| **MOLA1546** | *Roccella fuciformis* | Maritime | MA | Wash | 830 |
| **MOLA1558** | *Roccella fuciformis* | Maritime | MA | Homogenate | 830 |
| **MOLA1557** | *Roccella fuciformis* | Maritime | MA | Homogenate | 833 | *Mumia flava* | KC907394 | **96.88** | *Actinobacteria* | *Nocardioidaceae* |
| **MOLA1556** | *Roccella fuciformis* | Maritime | MA | Homogenate | 836 | *Microbacterium invictum* | AM949677 | 99.52 | *Actinobacteria* | *Microbacteriaceae* |
| **MOLA1550** | *Roccella fuciformis* | Maritime | MA | Wash | 836 |
| **MOLA1541** | *Roccella fuciformis* | Maritime | MA | Wash | 836 |
| **MOLA1571** | *Roccella fuciformis* | Maritime | MA | Homogenate | 836 |
| **MOLA1605** | *Roccella fuciformis* | Maritime | AIA | Wash | 838 | *Isoptericola nanjingensis* | HQ222356 | 98.77 | *Actinobacteria* | *Promicromonosporaceae* |
| **MOLA1532** | *Roccella fuciformis* | Maritime | MA | Wash | 838 |
| **MOLA1603** | *Roccella fuciformis* | Maritime | AIA | Homogenate | 825 | *Streptomyces drozdowiczii* | AB249957 | 99.51 | *Actinobacteria* | *Streptomycetaceae* |
| **MOLA1601** | *Roccella fuciformis* | Maritime | AIA | Homogenate | 845 | *Agromyces terreus* | EF363711 | 100.00 | *Actinobacteria* | *Microbacteriaceae* |
| **MOLA1598** | *Roccella fuciformis* | Maritime | MA | Homogenate | 845 |
| **MOLA1600** | *Roccella fuciformis* | Maritime | AIA | Homogenate | 830 | *Streptomyces atroolivaceus* | AJ781320 | 99.63 | *Actinobacteria* | *Streptomycetaceae* |
| **MOLA1610** | *Roccella fuciformis* | Maritime | AIA | Wash | 830 |
| **MOLA1606** | *Roccella fuciformis* | Maritime | MA | Wash | 774 | *Microbacterium sacharophilum* | AB736273 | 98.45 | *Actinobacteria* | *Microbacteriaceae* |
| **MOLA1617** | *Roccella fuciformis* | Maritime | AIA | Homogenate | 664 | *Streptomyces albiaxialis* | AY999901 | 99.25 | *Actinobacteria* | *Streptomycetaceae* |
| **MOLA1616** | *Roccella fuciformis* | Maritime | AIA | Homogenate | 835 | *SEdiminihabitans luteus* | AB695376 | 99.27 | *Actinobacteria* | *Sanguibacteraceae* |
| **MOLA1607** | *Roccella fuciformis* | Maritime | AIA | Wash | 845 | *Nocardioides albus* | AF004988 | 99.76 | *Actinobacteria* | *Nocardioidaceae* |
| **MOLA1568** | *Roccella fuciformis* | Maritime | MA | Homogenate | 829 | *Brevibacterium epidermidis* | X76565 | 99.63 | *Actinobacteria* | *Brevibacteriaceae* |
| **DP_00094** | *Collema auriforme* | Inland | ISP2 | Homogenate | 874 | *Nocardia ignorata* | AJ303008 | 98.59 | *Actinobacteria* | *Nocardioidaceae* |
| **DP_00088** | *Collema auriforme* | Inland | ISP2 | Homogenate | 930 | *Bacillus anthracis* | AB190217 | 98.80 | *Firmicutes* | *Bacillaceae* |
| **DP_00085** | *Collema auriforme* | Inland | ISP2 | Homogenate | 870 | *Streptomyces xanthophaeus* | AB184177 | 99.05 | *Actinobacteria* | *Streptomycetaceae* |

| **DP_00083** | *Collema auriforme* | Inland | ISP2 | Homogenate | 917 | *Pseudomonas mohnii* | AM293567 | 99.18 | *Gammaproteobacteria* | *Pseudomonadaceae* |
| --- | --- | --- | --- | --- | --- | --- | --- | --- | --- | --- |
| **DP_00082** | *Collema auriforme* | Inland | ISP2 | Homogenate | 899 | *Arthrobacter nicotinovorans* | X80743 | 97.79 | *Actinobacteria* | *Micrococcaceae* |
| **DP_00079** | *Collema auriforme* | Inland | ISP2 | Homogenate | 894 | *Pseudomonas mohnii* | AM293567 | 99.18 | *Gammaproteobacteria* | *Pseudomonadaceae* |
| **DP_00063** | *Collema auriforme* | Inland | ISP2 | Wash | 822 | *Streptomyces xanthophaeus* | AB184177 | **95.94** | *Actinobacteria* | *Streptomycetaceae* |
| **DP_00061** | *Collema auriforme* | Inland | ISP2 | Wash | 906 | *Chryseobacterium aquaticum* | AM748690 | **96.71** | *Alphaproteobacteria* | *Flavobacteriaceae* |
| **DP_00060** | *Collema auriforme* | Inland | ISP2 | Wash | 905 | *Pseudoduganella violaceinigra* | AY376163 | 97.14 | *Betaproteobacteria* | *Oxalobacteraceae* |
| **DP_00054** | *Collema auriforme* | Inland | ISP2 | Wash | 944 | *Bacillus anthracis* | AB190217 | 98.32 | *Firmicutes* | *Bacillaceae* |
| **DP_00029** | *Collema auriforme* | Inland | ISP2 | Wash | 905 | *Pseudomonas arsenicoxydans* | FN645213 | 99.20 | *Gammaproteobacteria* | *Pseudomonadaceae* |
| **DP_00015** | *Collema auriforme* | Inland | ISP2 | Wash | 915 | *Streptomyces spororaveus* | AJ781370 | 98.03 | *Actinobacteria* | *Streptomycetaceae* |
| **DP_00007** | *Collema auriforme* | Inland | ISP2 | Wash | 842 | *Sphingomonas cynarae* | HQ439186 | 97.78 | *Alphaproteobacteria* | *Sphingomonadaceae* |
| **DP_00004** | *Collema auriforme* | Inland | ISP2 | Wash | 848 | *Sphingomonas aquatilis* | AF131295 | 98.28 | *Alphaproteobacteria* | *Sphingomonadaceae* |
| **DP_00001** | *Collema auriforme* | Inland | ISP2 | Wash | 876 | *Streptomyces spororaveus* | AJ781370 | 98.59 | *Actinobacteria* | *Streptomycetaceae* |
| **DP_00095** | *Collema auriforme* | Inland | ISP2 | Homogenate | 822 | *Nocardia ninae* | DQ235687 | 99.25 | *Actinobacteria* | *Nocardioidaceae* |
| **DP_00093** | *Collema auriforme* | Inland | ISP2 | Homogenate | 842 | *Massilia namucuonensis* | JF799985 | **96.91** | *Betaproteobacteria* | *Oxalobacteraceae* |
| **DP_00043** | *Collema auriforme* | Inland | ISP2 | Wash | 842 |  |  |  |  |  |
| **DP_00092** | *Collema auriforme* | Inland | ISP2 | Homogenate | 837 | *Agromyces terreus* | EF363711 | 99.04 | *Actinobacteria* | *Microbacteriaceae* |
| **DP_00081** | *Collema auriforme* | Inland | ISP2 | Homogenate | 854 | *Pseudomonas mohnii* | AM293567 | 99.88 | *Gammaproteobacteria* | *Pseudomonadaceae* |
| **DP_00080** | *Collema auriforme* | Inland | ISP2 | Homogenate | 829 | *Variovorax ginsengisoli* | AB254358 | **96.98** | *Betaproteobacteria* | *Comamonadaceae* |
|  |  |  |  |  |  |  | Y18616 |  |  |  |
| **DP_00075** | *Collema auriforme* | Inland | ISP2 | Homogenate | 853 | *Acidovorax defluvii* |  | 99.06 | *Betaproteobacteria* | *Comamonadaceae* |
| **DP_00020** | *Collema auriforme* | Inland | ISP2 | Wash | 853 |  |  |  |  |  |
| **DP_00051** | *Collema auriforme* | Inland | ISP2 | Wash | 853 |  |  |  |  |  |
| **DP_00073** | *Collema auriforme* | Inland | ISP2 | Homogenate | 796 | *Caulobacter henricii* | AJ227758 | 98.99 | *Alphaproteobacteria* | *Caulobacteraceae* |
| **DP_00072** | *Collema auriforme* | Inland | ISP2 | Homogenate | 796 |  |  |  |  |  |
| **DP_00071** | *Collema auriforme* | Inland | ISP2 | Homogenate | 850 | *Pseudomonas mandelii* | AF058286 | 99.29 | *Gammaproteobacteria* | *Pseudomonadaceae* |
| **DP_00032** | *Collema auriforme* | Inland | ISP2 | Wash | 850 |  |  |  |  |  |
| **DP_00066** | *Collema auriforme* | Inland | ISP2 | Homogenate | 838 | *Pedobacter roseus* | DQ112353 | 100.00 | *Alphaproteobacteria* | *Sphingobacteriaceae* |
| **DP_00062** | *Collema auriforme* | Inland | ISP2 | Wash | 790 | *Rhizobium rubi* | D14503 | 99.11 | *Alphaproteobacteria* | *Rhizobiaceae* |
| **DP_00047** | *Collema auriforme* | Inland | ISP2 | Wash | 838 | *Pseudomonas mohnii* | AM293567 | 100.00 | *Gammaproteobacteria* | *Pseudomonadaceae* |
| **DP_00045** | *Collema auriforme* | Inland | ISP2 | Wash | 863 | *Pseudomonas frederiksbergensis* | AJ249382 | 99.52 | *Gammaproteobacteria* | *Pseudomonadaceae* |
| **DP_00044** | *Collema auriforme* | Inland | ISP2 | Wash | 822 | *Arthrobacter nicotinovorans* | X80743 | 99.88 | *Actinobacteria* | *Micrococcaceae* |
|  |  |  |  |  |  |  |  |  |  |  |

| **DP_00038** | *Collema auriforme* | Inland | ISP2 | Wash | 848 | *Pseudomonas jessenii* | AF068259 | 99.64 | *Gammaproteobacteria* | *Pseudomonadaceae* |
| --- | --- | --- | --- | --- | --- | --- | --- | --- | --- | --- |
| **DP_00052** | *Collema auriforme* | Inland | ISP2 | Wash | 846 | *Pseudomonas mohnii* | AM293567 | 99.76 | *Gammaproteobacteria* | *Pseudomonadaceae* |
| **DP_00074** | *Collema auriforme* | Inland | ISP2 | Homogenate | 846 |
| **DP_00069** | *Collema auriforme* | Inland | ISP2 | Homogenate | 846 |
| **DP_00070** | *Collema auriforme* | Inland | ISP2 | Homogenate | 846 |
| **DP_00046** | *Collema auriforme* | Inland | ISP2 | Wash | 846 |
| **DP_00013** | *Collema auriforme* | Inland | ISP2 | Wash | 846 |
| **DP_00003** | *Collema auriforme* | Inland | ISP2 | Wash | 846 |
| **DP_00033** | *Collema auriforme* | Inland | ISP2 | Wash | 846 |
| **DP_00048** | *Collema auriforme* | Inland | ISP2 | Wash | 846 |
| **DP_00025** | *Collema auriforme* | Inland | ISP2 | Wash | 828 | *Rhodococcus erythropolis* | X79289 | 99.76 | *Actinobacteria* | *Micrococcaceae* |
| **DP_00024** | *Collema auriforme* | Inland | ISP2 | Wash | 871 | *Bacillus weihenstephanensis* | BAUY01000093 | 99.89 | *Firmicutes* | *Bacillaceae* |
| **DP_00087** | *Collema auriforme* | Inland | ISP2 | Homogenate | 871 |
| **DP_00016** | *Collema auriforme* | Inland | ISP2 | Wash | 871 |
| **DP_00097** | *Collema auriforme* | Inland | ISP2 | Homogenate | 871 |
| **DP_00053** | *Collema auriforme* | Inland | ISP2 | Wash | 871 |
| **DP_00023** | *Collema auriforme* | Inland | ISP2 | Wash | 808 | *Sphingomonas cynarae* | HQ439186 | 98.49 | *Alphaproteobacteria* | *Sphingomonadaceae* |
| **DP_00005** | *Collema auriforme* | Inland | ISP2 | Wash | 808 |  |  |  |
| **DP_00022** | *Collema auriforme* | Inland | ISP2 | Wash | 841 | *Duganella radicis* | EU672807 | **96.58** | *Betaproteobacteria* | *Oxalobacteraceae* |
| **DP_00018** | *Collema auriforme* | Inland | ISP2 | Wash | 799 | *Caulobacter henricii* | AJ227758 | 99.37 | *Alphaproteobacteria* | *Caulobacteraceae* |
| **DP_00011** | *Collema auriforme* | Inland | ISP2 | Wash | 863 | *Pseudomonas helmanticensis* | HG940537 | 99.76 | *Gammaproteobacteria* | *Pseudomonadaceae* |
| **DP_00049** | *Collema auriforme* | Inland | ISP2 | Wash | 863 |
| **DP_00058** | *Collema auriforme* | Inland | ISP2 | Wash | 863 |
| **DP_00030** | *Collema auriforme* | Inland | ISP2 | Wash | 863 |
| **DP_00006** | *Collema auriforme* | Inland | ISP2 | Wash | 807 | *Sphingomonas cynarae* | HQ439186 | 98.49 | *Gammaproteobacteria* | *Sphingomonadaceae* |
| **DP_00002** | *Collema auriforme* | Inland | ISP2 | Wash | 845 | *Pedobacter kyungheensis* | JN196132 | 97.98 | *Alphaproteobacteria* | *Sphingobacteriaceae* |
| **DP_00041**  **DP_00042** | *Collema auriforme*  *Collema auriforme* | Inland  Inland | ISP2  ISP2 | Wash  Wash | 859  859 | *‘Chryseobacterium yeoncheonense’* | JX141782 | 97.98 | *Alphaproteobacteria* | *Flavobacteriaceae* |

**Table S2**: Classification of OTUs based in the RDP_classifier identification (0.8 probability) of 16S rRNA sequences. p__: phylum. c__: class. f__ : family. g__: genus. s__: species

| OTU ID | Taxonomy | |
| --- | --- | --- |
| 24 | Root; p__*Proteobacteria*; c__*Alphaproteobacteria*; f__*Rhodobacteraceae* | |
| 25 | Root; p__*Actinobacteria*; c__*Actinobacteria*; f__*Nocardioidaceae*; g__; s__ | |
| 26 | Root; p__*Actinobacteria*; c__*Actinobacteria*; f__*Microbacteriaceae*; g__*Microbacterium* | |
| 27 | Root; p__*Actinobacteria*; c__*Actinobacteria*; f__*Promicromonosporaceae*; g__*Isoptericola*; s__ | |
| 21 | Root; p__*Actinobacteria*; c__*Actinobacteria*; f__Micrococcaceae; g__*Micrococcus*; s__luteus | |
| 22 | Root; p__*Proteobacteria*; c__*Alphaproteobacteria*; f__*Rhodobacteraceae* | |
| 28 | Root; p__*Actinobacteria*; c__*Actinobacteria*; f__*Pseudonocardiaceae*; g__*Saccharopolyspora*; s__ | |
| 29 | Root; p__*Proteobacteria*; c__*Alphaproteobacteria*; f__*Rhodobacteraceae*; g__*Paracoccus*; s__*aminovorans* | |
| 8 | Root; p__*Proteobacteria*; c__*Alphaproteobacteria*; f__*Rhodobacteraceae*; g__*Paracoccus* | |
| 59 | Root; p__*Firmicutes*; c__*Bacilli*; f__*Bacillaceae*; g__; s__ | |
| 58 | Root; p__*Proteobacteria*; c__*Alphaproteobacteria*; f__*Erythrobacteraceae*; g__; s__ | |
| 55 | Root; p__*Actinobacteria*; c__*Actinobacteria*; f__*Gordoniaceae*; g__*Gordonia*; s__ | |
| 54 | Root; p__*Actinobacteria*; c__*Actinobacteria*; f__*Mycobacteriaceae*; g__*Mycobacterium*; s__ | |
| 57 | Root; p__*Proteobacteria*; c__*Alphaproteobacteria*; f__Erythrobacteraceae | |
| 51 | Root; p__*Firmicutes*; c__*Bacilli*; f__*Planococcaceae*; g__*Planococcus* | |
| 50 | Root; p__*Proteobacteria*; c__*Alphaproteobacteria*; f__*Erythrobacteraceae* | |
| 53 | Root; p__*Actinobacteria*; c__*Actinobacteria*; f__*Micrococcaceae* | |
| 52 | Root; p__*Actinobacteria*; c__*Actinobacteria*; f__*Streptomycetaceae*; g__; s__ | |
| 89 | Root; p__*Actinobacteria*; c__*Actinobacteria*; f__*Microbacteriaceae*; g__*Microbacterium* | |
| 83 | Root; p__*Actinobacteria*; c__*Actinobacteria*; f__*Streptomycetaceae*; g__*Streptomyces* | |
| 87 | Root; p__*Actinobacteria*; c__*Actinobacteria*; f__*Microbacteriaceae*; g__*Microbacterium*; s__ | |
| 84 | Root; p__*Actinobacteria*; c__*Actinobacteria*; f__*Mycobacteriaceae*; g__*Mycobacterium*; s__*vaccae* | |
| 85 | Root; p__*Firmicutes*; c__*Bacilli*; f__*Bacillaceae*; g__*Bacillus*; s__*muralis* | |
| 3 | Root; p__*Proteobacteria*; c__*Alphaproteobacteria* | |
| 7 | Root; p__*Proteobacteria*; c__*Alphaproteobacteria*; f__Sphingomonadaceae; g__; s__ | |
| 100 | Root; p__*Actinobacteria*; c__*Actinobacteria*; f__*Brevibacteriaceae*; g__*Brevibacterium*; s__ | |
| 39 | Root; p__*Proteobacteria*; c__*Alphaproteobacteria*; f__*Rhodobacteraceae*; g__; s__ | |
|  | |  |
|  | |  |
| **Table S2 (continued)** | | |
| 38 | Root; p__*Actinobacteria*; c__*Actinobacteria*; f__*Microbacteriaceae*; g__*Agromyces*; s__ | |
| 33 | Root; p__*Firmicutes*; c__*Bacilli*; f__*Staphylococcaceae*; g__*Staphylococcus*; s__*epidermidis* | |
| 32 | Root; p__*Actinobacteria*; c__*Actinobacteria*; f__*Gordoniaceae*; g__*Gordonia*; s__ | |
| 31 | Root; p__*Actinobacteria*; c__*Actinobacteria*; f__*Streptomycetaceae*; g__*Streptomyces*; s__ | |
| 30 | Root; p__*Firmicutes*; c__*Bacilli*; f__*Bacillaceae*; g__*Bacillus*; s__ | |
| 37 | Root; p__*Actinobacteria*; c__*Actinobacteria*; f__*Microbacteriaceae*; g__*Microbacterium*; s__*aurum* | |
| 36 | Root; p__*Actinobacteria*; c__*Actinobacteria*; f__*Microbacteriaceae*; g__*Microbacterium* | |
| 61 | Root; p__*Proteobacteria*; c__*Alphaproteobacteria*; f__*Phyllobacteriaceae*; g__; s__ | |
| 62 | Root; p__*Proteobacteria*; c__*Alphaproteobacteria*; f__*Rhodobacteraceae*; g__*Anaerospora*; s__*hongkongensis* | |
| 63 | Root; p__*Proteobacteria*; c__*Alphaproteobacteria*; f__*Erythrobacteraceae* | |
| 64 | Root; p__*Proteobacteria*; c__*Alphaproteobacteria*; f__*Sphingomonadaceae*; g__; s__ | |
| 65 | Root; p__*Proteobacteria*; c__*Alphaproteobacteria*; f__*Rhodobacteraceae*; g__; s__ | |
| 66 | Root; p__*Proteobacteria*; c__*Alphaproteobacteria*; f__*Erythrobacteraceae*; g__; s__ | |
| 67 | Root; p__*Proteobacteria*; c__*Alphaproteobacteria*; f__*Caulobacteraceae*; g__*Phenylobacterium*; s__ | |
| 68 | Root; p__*Proteobacteria*; c__*Alphaproteobacteria*; f__*Sphingomonadaceae*; g__; s__ | |
| 2 | Root; p__*Actinobacteria*; c__*Actinobacteria*; f__*Cellulomonadaceae*; g__; s__ | |
| 6 | Root; p__*Actinobacteria*; c__*Actinobacteria*; f__*Gordoniaceae*; g__*Gordonia*; s__ | |
| 99 | Root; p__*Actinobacteria*; c__*Actinobacteria*; f__*Nocardioidaceae*; g__; s__ | |
| 98 | Root; p__*Actinobacteria*; c__*Actinobacteria*; f__*Microbacteriaceae*; g__*Microbacterium*; s__ | |
| 91 | Root; p__*Actinobacteria*; c__*Actinobacteria*; f__*Brevibacteriaceae*; g__*Brevibacterium*; s__*aureum* | |
| 93 | Root; p__*Actinobacteria*; c__*Actinobacteria*; f__*Microbacteriaceae*; g__*Salinibacterium*; s__*amurskyense* | |
| 92 | Root; p__*Actinobacteria*; c__*Actinobacteria*; f__*Nocardioidaceae*; g__*Aeromicrobium*; s__ | |
| 95 | Root; p__*Actinobacteria*; c__*Actinobacteria*; f__*Micrococcaceae*; g__*Kocuria*; s__*rhizophila* | |
| 94 | Root; p__*Actinobacteria*; c__*Actinobacteria*; f__*Promicromonosporaceae* | |
| 97 | Root; p__*Bacteroidetes*; c__*Sphingobacteriia*; f__*Saprospiraceae*; g__*Lewinella*; s__ | |
| 96 | Root; p__*Actinobacteria*; c__*Actinobacteria*; f__*Microbacteriaceae*; g__*Curtobacterium*; s__ | |
| 11 | Root; p__*Actinobacteria*; c__*Actinobacteria*; f__*Cellulomonadaceae*; g__; s__ | |
| 13  12 | Root; p__*Actinobacteria*; c__*Actinobacteria*; f__*Microbacteriaceae*; g__*Microbacterium*  Root; p__*Actinobacteria*; c__*Actinobacteria*; f__*Microbacteriaceae*; g__*Microbacterium*; s__ | |
|  |  | |
|  | | |
|  | | |
| **Table S2 (Continued)** | | |
| 15 | Root; p__*Firmicutes*; c__*Bacilli*; f__*Bacillaceae*; g__*Bacillus*; s__*baekryungensis* | |
| 14 | Root; p__*Proteobacteria*; c__*Alphaproteobacteria*; f__*Sphingomonadaceae*; g__; s__ | |
| 17 | Root; p__*Actinobacteria*; c__*Actinobacteria*; f__*Cellulomonadaceae*; g__; s__ | |
| 19 | Root; p__*Actinobacteria*; c__*Actinobacteria*; f__*Brevibacteriaceae*; g__*Brevibacterium*; s__*aureum* | |
| 18 | Root; p__*Actinobacteria*; c__*Actinobacteria*; f__*Nocardioidaceae*; g__; s__ | |
| 48 | Root; p__*Firmicutes*; c__*Bacilli*; f__*Bacillaceae*; g__; s__ | |
| 49 | Root; p__*Proteobacteria*; c__*Alphaproteobacteria*; f__*Sphingomonadaceae*; g__; s__ | |
| 46 | Root; p__*Actinobacteria*; c__*Actinobacteria*; f__*Microbacteriaceae*; g__*Microbacterium* | |
| 47 | Root; p__*Actinobacteria*; c__*Actinobacteria*; f__*Streptomycetaceae*; g__; s__ | |
| 44 | Root; p__*Actinobacteria*; c__*Actinobacteria*; f__*Streptomycetaceae*; g__; s__ | |
| 43 | Root; p__*Actinobacteria*; c__*Actinobacteria*; f__*Streptomycetaceae*; g__*Streptomyces* | |
| 40 | Root; p__*Actinobacteria*; c__*Actinobacteria*; f__*Microbacteriaceae*; g__*Agromyces*; s__ | |
| 1 | Root; p__*Actinobacteria*; c__*Actinobacteria*; f__*Streptomycetaceae*; g__*Streptomyces* | |
| 5 | Root; p__*Actinobacteria*; c__*Actinobacteria*; f__*Promicromonosporaceae*; g__*Cellulosimicrobium*; s__ | |
| 9 | Root; p__*Proteobacteria*; c__*Alphaproteobacteria*; f__*Erythrobacteraceae*; g__; s__ | |
| 75 | Root; p__*Firmicutes*; c__*Bacilli*; f__*Bacillaceae*; g__*Bacillus*; s__*flexus* | |
| 34 | Root; p__*Bacteroidetes*; c__*Flavobacteriia*; f__*Flavobacteriaceae*; g__*Chryseobacterium*; s__ | |
| 74 | Root; p__*Firmicutes*; c__*Bacilli*; f__*Bacillaceae*; g__*Bacillus*; s__ | |
| 73 | Root; p__*Firmicutes*; c__*Bacilli*; f__*Planococcaceae*; g__*Planococcus*; s__ | |
| 72 | Root; p__*Actinobacteria*; c__*Actinobacteria*; f__*Microbacteriaceae*; g__*Agromyces*; s__ | |
| 70 | Root; p__*Actinobacteria*; c__*Actinobacteria*; f__*Nocardioidaceae*; g__; s__ | |
